# Machine learning-driven identification of drugs inhibiting cytochrome P450 2C9

**DOI:** 10.1371/journal.pcbi.1009820

**Published:** 2022-01-26

**Authors:** Elodie Goldwaser, Catherine Laurent, Nathalie Lagarde, Sylvie Fabrega, Laure Nay, Bruno O. Villoutreix, Christian Jelsch, Arnaud B. Nicot, Marie-Anne Loriot, Maria A. Miteva

**Affiliations:** 1 INSERM U1268 « Medicinal Chemistry and Translational Research », UMR 8038 CiTCoM, CNRS—University of Paris, Paris, France; 2 University of Paris, INSERM U1138, Paris, France; 3 Laboratoire GBCM, EA7528, Conservatoire National des Arts et Métiers, 2 Rue Conté, Hésam Université, Paris, France; 4 Viral Vector for Gene Transfer core facility, Université de Paris—Structure Fédérative de Recherche Necker, INSERM US24/CNRS UMS3633, Paris, France; 5 INSERM UMR 1141, Robert-Debré Hospital, Paris, France; 6 CRM2, UMR CNRS 7036, Université de Lorraine, Nancy, France; 7 INSERM, Nantes Université, Center for Research in Transplantation and Translational Immunology, UMR 1064, ITUN, Nantes, France; 8 Assistance Publique-Hôpitaux de Paris, Hôpital Européen Georges Pompidou, Service de Biochimie, Paris, France; Icahn School of Medicine at Mount Sinai, UNITED STATES

## Abstract

Cytochrome P450 2C9 (CYP2C9) is a major drug-metabolizing enzyme that represents 20% of the hepatic CYPs and is responsible for the metabolism of 15% of drugs. A general concern in drug discovery is to avoid the inhibition of CYP leading to toxic drug accumulation and adverse drug–drug interactions. However, the prediction of CYP inhibition remains challenging due to its complexity. We developed an original machine learning approach for the prediction of drug-like molecules inhibiting CYP2C9. We created new predictive models by integrating CYP2C9 protein structure and dynamics knowledge, an original selection of physicochemical properties of CYP2C9 inhibitors, and machine learning modeling. We tested the machine learning models on publicly available data and demonstrated that our models successfully predicted CYP2C9 inhibitors with an accuracy, sensitivity and specificity of approximately 80%. We experimentally validated the developed approach and provided the first identification of the drugs vatalanib, piriqualone, ticagrelor and cloperidone as strong inhibitors of CYP2C9 with IC values <18 μM and sertindole, asapiprant, duvelisib and dasatinib as moderate inhibitors with IC50 values between 40 and 85 μM. Vatalanib was identified as the strongest inhibitor with an IC50 value of 0.067 μM. Metabolism assays allowed the characterization of specific metabolites of abemaciclib, cloperidone, vatalanib and tarafenacin produced by CYP2C9. The obtained results demonstrate that such a strategy could improve the prediction of drug-drug interactions in clinical practice and could be utilized to prioritize drug candidates in drug discovery pipelines.

## Author summary

Cytochrome P450 (CYP) is a superfamily of heme-containing oxidizing enzymes responsible for the metabolism of a wide variety of drugs, xenobiotics and endogenous molecules. Five of the human CYPs (1A2, 2C9, 2C19, 2D6, and 3A4) are involved in ∼95% of the CYP-mediated metabolism of drugs representing ∼75% of drug metabolism. CYP inhibition leads to decreased drugs/chemicals elimination, which is a major cause of drug-drug interactions provoking adverse drug reactions. We developed an original integrated structure-based and machine learning approach for the prediction of CYP2C9 inhibitors. It exhibited excellent performance and its application allowed to demonstrate for the first time that the drugs vatalanib, piriqualone, ticagrelor and cloperidone are strong inhibitors of CYP2C9.

This is a *PLOS Computational Biology* Methods paper.

## Introduction

Cytochrome P450 (CYP) is a superfamily of heme-containing oxidizing enzymes responsible for the metabolism of a wide variety of drugs, xenobiotics and endogenous molecules [1–4]. Five of the human CYPs (1A2, 2C9, 2C19, 2D6, and 3A4) are involved in ∼95% of the CYP-mediated metabolism of drugs representing ∼75% of drug metabolism [5]. In addition, the large contributions of the CYPs 3A4 and 2C9 are driven to a large extent by the high expression levels of these two enzymes in the human liver and intestine and to their broad substrate specificity [6]. A general concern in drug discovery is avoiding the inhibition of drug-metabolizing CYPs. CYP inhibition can lead to decreased drugs/chemicals elimination, which is a major cause of drug-drug interactions (DDI) provoking severe adverse events [3,7,8]. Therefore, identifying the potential inhibition of CYP is critical for drug development and clinical drug treatment.

Numerous computational approaches have been developed attempting to predict CYP-mediated metabolism and inhibition [9–11]. The publicly accessible data accumulated through academic bioassays (e.g., PubChem and ChEMBL) have enabled the development of quantitative structure-activity relationship (QSAR) models for the *in silico* prediction of CYP inhibition and of multiple-category classification models for several CYP isoforms using machine learning (ML) methods [12–14]. These models have used structural rules [15] or molecular descriptors of CYP inhibitors without considering their interactions with the protein structure. Overall, such models show good predictive performances, but employing mechanistic knowledge and the 3D structure of this family of enzymes has been demonstrated to be very useful [16–19]. For example, Joshi et al [20] identified potent and selective CYP1A1 inhibitors via combined ligand-based pharmacophore and structure-based virtual screening. Further, the numerous experimental 3D structures of CYP bound to various substrates and inhibitors available in the PDB [21] demonstrate that the CYP active site is extremely plastic and accommodates structurally diverse ligands of different size [22,23]. Therefore, the flexibility of CYP plays an important role in these interactions [24,25]. Previously, we developed a machine learning approach for the prediction of CYP2D6 inhibition by combining ligand-based extended connectivity fingerprints and ligand interaction energies, as different models were built corresponding to different CYP2D6 conformations [26].

Here, we developed an original approach based on machine learning that allows prediction of inhibitors of CYP2C9. This enzyme represents approximately 20% of the total hepatic CYPs and metabolizes more than 15% of clinically administered drugs [22,27,28] and several endogenous compounds [29]. Recently, we have explored a large region of the conformational space of CYP2C9 in apo and substrate-bound states using molecular dynamics (MD) simulations combined with energetic analyses, which allowed us to generate protein conformations representing key movements of the binding site [30]. Here, we built novel predictive models by combining CYP2C9 protein structure and dynamics knowledge, an original selection of physicochemical descriptors of CYP2C9 inhibitors, and ML support vector machine (SVM) and random forest (RF) algorithms. The validation on PubChem Bioassay and ChEMBL data demonstrated that developed SVM and RF models successfully classified inhibitors and non-inhibitors of CYP2C9. The ML models were then used to screen 4480 experimental and approved drugs. Eighteen chemically diverse drugs were subjected to *in vitro* CYP2C9 inhibition tests, and metabolism assays were additionally performed for some of them. We demonstrated for the first time that the drugs vatalanib, piriqualone, ticagrelor and cloperidone are strong inhibitors of CYP2C9 and specific metabolites of abemaciclib, cloperidone, vatalanib and tarafenacin are produced by CYP2C9.

## Results and discussion

### Integrated structure-based and machine learning modeling to predict inhibition of CYP2C9

**Fig 1:**
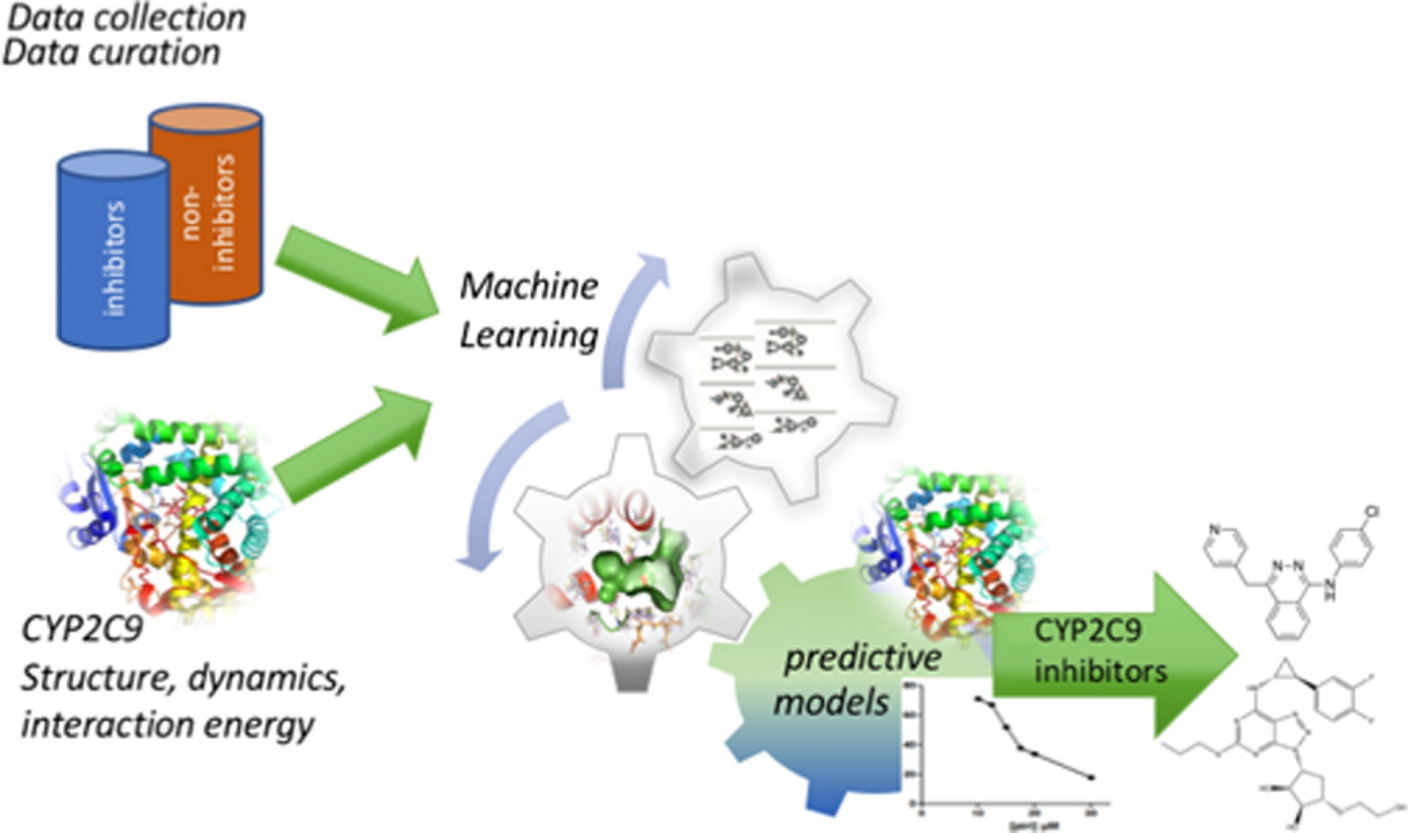
Workflow of this research that includes datasets preparation, CYP2C9 structure, dynamics and ligand binding analyses, machine learning to train different predictive models and *in vitro* identification of new drug inhibitors of CYP2C9.

In this study, we developed classification machine learning models for the prediction of CYP2C9 inhibition and for the identification of new drugs inhibiting CYP2C9 (Fig 1). We collected known inhibitors and non-inhibitors of CYP2C9 from the ChEMBL [31] and PubChem databases [32] to build training and external test datasets for ML modeling. In order to create predictive models with applicability covering drug-like molecules while maintaining chemical diversity we performed “soft” drug-like filtering [33] and diversity clustering of the collected compounds (see *Methods* for details). The filtered dataset of the diverse clusters centroids contained 4840 inhibitors and 3301 non-inhibitors of CYP2C9. Among them, we randomly selected 80% of the active and inactive compounds for the training set and the remaining 20% constituted the external validation test set while conserving the same proportion of inhibitors and non-inhibitors. The plots of molecular weight (MW) *vs*. logP of the training and external test sets’ inhibitors and non-inhibitors are shown in S1 Fig. Overall, MW and logP of the training and test sets’ compounds are within the same ranges. Our models are applicable within a domain given by the “soft” drug-like filter thresholds (see in S1 Text).

To consider the CYP2C9 flexibility, we took into account conformational changes of CYP2C9 based on our recent MD simulations [30]. Those MD simulations of 750 ns explored a large region of the conformational space of the wild-type of CYP2C9 in apo or substrate-bound states for the active species of the enzyme with the heme present in the Compound I state (Cpd I), which is consistent with the catalytic reaction. The drugs diclofenac and losartan, typical substrates of CYP2C9, were present during the MD simulations in the substrate-bound states. In that work [30], we performed structural clustering using with a Root Mean Square Deviation (RMSD) distance of at least 1.0 Å for all atoms of the binding site. The most populated 40 clusters (corresponding to the conformations covering 85% of the MD simulations) generated for each of the three studied systems, CYP2C9 apo, CYP2C9 diclofenac-bound, CYP2C9 losartan-bound, were retained. Then, molecular docking of five diverse drugs, known substrates of CYP2C9, warfarin, diclofenac, glimepiride, flurbiprofen and losartan, was performed into the MD conformational ensemble of the retained 120 centroids and into several crystal structures of CYP2C9. These docking analyses allowed us to identify the best five MD protein conformations (MD1, MD2, MD3, MD4 and MD5) with a competent substrate orientation in the active site and two crystal structures (PDB IDs 1R9O, 5XXI) [34,35] (see S2 Fig and S1 Table) as representative conformations for key movements of the binding pocket area [30]. The seven chosen CYP2C9 structures showed important conformational changes of the binding site allowing to accommodate diverse ligands. Here, the training set of the diverse CYP2C9 inhibitors and non-inhibitors were docked into these two crystal and five MD protein structures using AutoDock Vina software [36] (see *Methods* for details). The computed interaction energies (IEs) by docking-scoring (shown in S3 Fig) were then used (1) as descriptors for the machine learning models, and (2) as an additional filter for predicting CYP2C9 inhibitors.

Physicochemical molecular descriptors of the training set’s molecules were calculated using MOE software [37]. Initially, we calculated 354 2D and 3D MOE descriptors. Highly correlated descriptors with an absolute value of the Pearson correlation coefficient greater than or equal to 0.85 and descriptors with near null variance were removed. This selection resulted in 170 descriptors. The calculated IEs for the seven CYP2C9 structures were added as structure-based descriptors accounting for the protein-ligand interactions. Then, to avoid overfitting and to propose an *in silico* strategy with a reduced calculation time, we selected the best descriptors based on their relative importance in predicting the inhibiting character of a molecule. That selection comprised building a plurality of RF models on the training dataset and selecting the subset of descriptors with the highest Gini importance [38]. The Gini index, also known as the Gini impurity index, is a measure of the probability of incorrectly classifying a randomly selected element in a dataset if it was randomly labeled according to the class distribution in the dataset. Thus, we ran RF computations with the 170 MOE and 7 IE descriptors by performing a scan over the *ntree* and *mtry* parameters (see *Methods* for details). Then, keeping the parameters with the best performance, we performed 2000 RF runs to calculate the mean importance of the 177 descriptors according to the diminution of the Gini criterion (S4 Fig). Following the importance of the descriptors calculated (shown in S4 Fig), the first 10 descriptors (including only MOE descriptors) were critical for the model performance. The importance decreased slowly between the first 10 descriptors and the first 43 descriptors including 36 MOE and the 7 IEs. Thus, we built preliminary RF models with the best 10, 15, 20, 30 and 40 MOE descriptors in order to find the best combination of descriptors. Their performance (S2 Table) showed that starting from the 15 best MOE descriptors, good internal accuracy, sensitivity and specificity (> 75%) were achieved. Therefore, we further considered the best 15 and 20 MOE descriptors. In addition, the first 43 descriptors, including the 7 IE descriptors and showing an importance value > 20, were selected for further analyses (S1 Table). A plateau was observed (S4 Fig) for importance < 20, thus taking more descriptors would add a noise in the models. The seven IEs showed importance > 20 and two MD conformations and one crystal structure (PDB ID: 1R9O) showed importance > 30. The 36 best MOE descriptors in terms of importance can be seen in S1 Table. The most important descriptors corresponded to lipophilicity (2D), the number of aromatic bonds (2D), partial charges (2D), a negative van der Waals surface area (2D, 3D), and shape (3D: first diagonal element of diagonalized moment of inertia tensor, out-of-plane potential energy, and surface roughness). It appears that lipophilicity and solvent-exposed atoms with negative partial charges strongly contribute to the inhibition of CYP2C9. A major advantage of this approach compared to our previous one developed for the prediction of CYP2D6 inhibition [26] is that here a rational selection of the descriptors according to their importance is performed permitting thus to decrease the noise due to descriptors not sufficiently discriminating between the active and inactive compounds, and to reduce the computational time.

**Fig 2:**
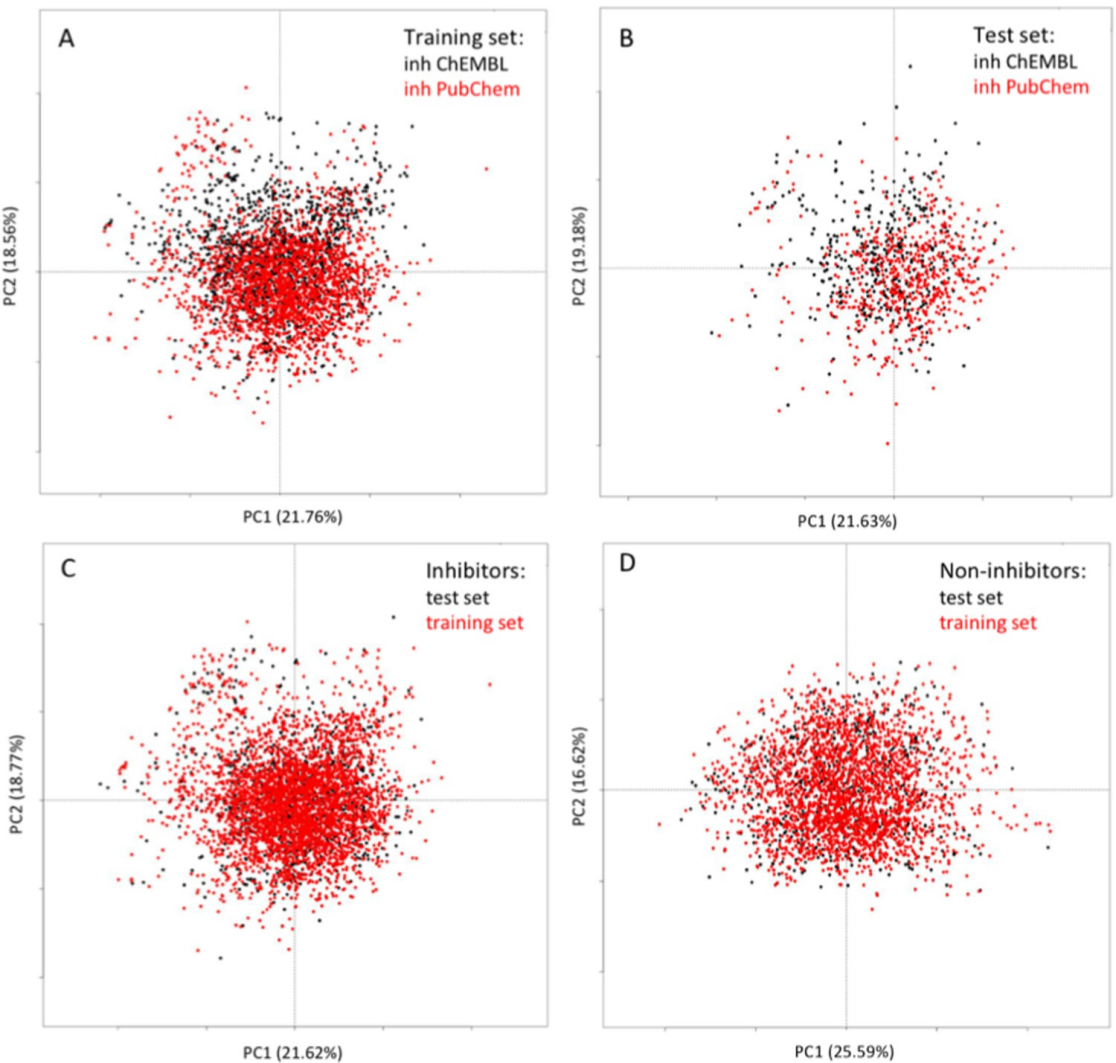
Chemical space of the training and test sets as described by the principal component analysis (PCA). The first two components, and their representation in % of the total variance are shown. (A). PCA of the training set’ inhibitors of PubChem *vs*. ChEMBL data sets. (B) PCA of the external test set’ inhibitors of PubChem *vs*. ChEMBL data sets. (C) PCA of the training and external test sets’ inhibitors. (D) PCA of the training and external test sets’ non-inhibitors.

Fig 2 represents the chemical space of the training and external test sets. The 36 best MOE descriptors were used to perform principal component analysis (PCA). The first two components were used for plotting. It is seen that the inhibitors taken from the ChEMBL data set shared the same chemical space as the PudChem ones, however, the ChEMBL data increased the diversity of the training and test sets’ inhibitors (Fig 2A and 2B). The inhibitors and non-inhibitors covered different chemical space (Figs 2C and 2D and S1).

### Performance of the predictive models on training and external test data

Then, we built the following RF and SVM models (see *Methods* for details) using: the 170 MOE + 7 IE descriptors, the 36 best MOE + 7 IE descriptors, 20 best MOE + 7 IE descriptors, and 15 best MOE + 7 IE descriptors. Cross-validation was applied for RF and SVM modeling. For RF modeling, the diversity on the training data set is achieved by use of multiple decision trees built with bootstrap samples from the training data and a small subset of descriptors randomly selected to make decisions at each node of the trees. The hyperparameters of the best trained RF and SVM models are shown in S3 Table. The performance of the models was assessed based on their accuracy, sensitivity, specificity and Matthews correlation coefficient (MCC) (see in S1 Text). The resulting performances of the best RF and SVM models created for different numbers of MOE descriptors and applied on the training and external validation test sets are summarized in Tables 1 and 2, respectively. The results showed that all the RF and SVM models have good predictive powers, and all the models succeeded to positively discriminate CYP2C9 inhibitors. All the models presented excellent sensitivities from 87% to 90% and high specificities ranging from 74% to 79% for the test set. For all the models, the sensitivity was higher than the specificity, which indicates the reliability of the detection of CYP2C9 inhibitors. For RF models training, the best results were obtained with the model including 36 MOE and 7 IE descriptors, as demonstrated by the internal and external accuracies equal to 83.66% and 85.55%, respectively, which are comparable to the performance achieved with the model including 170 MOE and 7 IE descriptors (84.12% and 85.55%, respectively).

**Table 1:** Performances of the optimized RF models with MOE and IE descriptors on the training set (cross-validation CV) and the external validation set.

Descriptors	Validation	Accuracy %	Sensitivity %	Specificity %	MCC %
15 MOE+7 IE	Training CV External set	82.81 84.82	88.44 89.17	74.67 78.07	64.17 67.92
20 MOE+7 IE	Training CV External set	83.25 84.45	88.57 89.57	75.55 76.52	65.10 67.08
36 MOE+7 IE	Training CV External set	83.66 85.55	89.13 89.97	75.76 78.69	65.96 69.45
170 MOE+7 IE	Training CV External set	84.12 85.55	90.02 90.57	75.58 77.76	66.90 69.41

**Table 2:** Performances of the optimized SVM models with MOE and IE descriptors on the training set (cross-validation CV) and the external validation set.

Descriptors	Validation	Accuracy %	Sensitivity %	Specificity %	MCC %
15 MOE+7 IE	Training CV External set	81.81 83.90	86.93 89.97	74.40 74.49	62.07 65.85
20 MOE+7 IE	Training CV External set	81.90 83.72	87.21 88.77	74.21 75.89	62.24 65.54
36 MOE+7 IE	Training CV External set	82.55 84.76	87.68 89.87	75.13 76.83	63.62 67.72
170 MOE+7 IE	Training CV External set	84.30 86.22	88.89 90.87	77.66 79.00	67.30 70.85

The comparison of the SVM models created with different numbers of descriptors led to the same conclusions. The best results were achieved with the model including 170 MOE and 7 IE descriptors, which obtained internal and external accuracies of 84.3% and 86.22%, respectively. The internal and external accuracies of the model with 36 MOE and 7 IE descriptors were very similar, with values of 82.55% and 84.76%, respectively. The performance of the RF and SVM models shown in Tables 1 and 2 was slightly better than the performance of the corresponding models without including the 7 IEs (shown in S4 Table). Furthermore, the IEs prediction provides direct information about the interactions between a putative inhibitor and CYP2C9 at the atomic level. We successfully employed such an IE filter for the identification of new inhibitors of CYP2C9 (see below). For all models, the performance on the external test set was slightly better than the internal performance. Although the diversity was ensured between all molecules of the training and the external test sets with a maximal chemical similarity of 0.85, that may happen due to the random choice of the molecules for the external set, that was also observed in other recent ML modeling studies [39,40]. The excellent performance obtained with the 36 best MOE and 7 IE descriptors suggest that these RF and SVM models can be employed to find new CYP2C9 inhibitors. Therefore, we retained these two models for further analyses.

The balanced accuracy, sensitivity, specificity and MCC values of our two final RF and SVM models with the 36 best MOE and 7 IE descriptors were compared with models predicting CYP2C9 inhibition reported in previous studies [18,41–45]. A benchmark of different datasets used and models’ performances as reported in the literature is given in S5 Table. In order to directly compare the performance of our models with other recent ones, we show here the performance of our final models and two state-of-the arts models available at the web servers CYPlebrity [44] and ADMETlab [45] on our external validation test set of inhibitors and non-inhibitors (see Table 3). Overall, our models performed better than the others (Tables 3 and S5), and showed remarkably better sensitivity, which is critical to detect inhibitors. The differences between our models and CYPlebrity and ADMETlab ones are statistically highly significant as seen from Table 4. To ensure the reliability of our models, we performed experimental validation and we successfully identified new CYP2C9 drug inhibitors.

**Table 3:** Comparison of the performances of the final RF and SVM models and other recent models on the external validation set.

Models	Accuracy %	Sensitivity %	Specificity %	MCC %
CYPlebrity	75.49	70.91	82.58	52.23
ADMETlab	79.57	70.81	93.01	62.69
RF 36 MOE + 7 IE	84.33	89.97	78.69	69.45
SVM 36 MOE + 7 IE	83.35	89.87	76.83	67.72

**Table 4:** Statistical significance (*p*-values) of the accuracy hypothesis between our final RF and SVM models and other recent models on the external validation set.

Models	CYPlebrity	ADMETlab
RF 36 MOE + 7 IE	9.9067e-08	1.1387e-23
SVM 36 MOE + 7 IE	1.0181e-08	1.8054e-25

### Application of the predictive models for the identification of new drugs inhibiting CYP2C9

We employed the retained ML models to screen 4480 approved and experimental drugs collected from four drug databases [46] (see in the S1 Text for their preparation) to identify new drugs that inhibit CYP2C9. Drugs already experimentally proven to inhibit CYP2C9 were not considered here. Given the performances of the best RF and SVM models with 36 MOE + 7 IE descriptors, we decided to combine both methods to identify unknown drug inhibitors of CYP2C9. The MOE descriptors and the interaction energies for the seven CYP2C9 protein conformations were calculated for the 4480 drugs. The consensus of the best RF and SVM models resulted in 2139 common drugs predicted as inhibitors of CYP2C9. To identify the most potent CYP2C9 drug inhibitors, we applied an additional filter of IE < -8.5 kcal/mol for each protein conformation corresponding to 75% of the IE scores of the training set’ inhibitors calculated within the seven protein conformations (S3 Fig). Although the scoring function of Autodock Vina is only an approximation of the free binding energy such a range is widely accepted for the prediction of strong protein-ligand interaction energies [26,47]. A similar approach combining ensemble docking and pharmacophore was previously employed to identify new ligands of another drug metabolizing enzyme, sulfotransferase SULT1E1 [26,47]. Finally, 720 drugs satisfied the RF, SVM, and IE criteria and were prioritized as inhibitors of CYP2C9. The results of a recent large-scale screening against five CYP isoforms showed that the majority of compounds cross-inhibited several isoforms, whereas only 7% of the compounds did not inhibit any of the isoforms [48]. CYPs are susceptible to be inhibited by a large variety of compounds, and thus, the high number of drugs predicted here to inhibit CYP2C9 seems to be reasonable. To select diverse drugs for the *in vitro* validation, we performed chemical diversity clustering with MOE software using MACCS fingerprints and a Tanimoto similarity cutoff of 0.80. The resulted 109 drug centroids were additionally classified using DataWarrior software [49] with the FragFp structure descriptor and a similarity cutoff of 0.70. Twenty-four clusters were thus obtained, as two of them combined diverse drugs with a structural FragFp similarity below 0.50 (Cluster 3 contained 18 molecules, and Cluster 24 contained 37 molecules).

### *In vitro* assays of CYP2C9 enzyme inhibition

The inhibition assays were performed with 18 candidate molecules (Table 5) selected from the 24 diversity classes after *in silico* screening. To obtain a representative selection of potential inhibitors and substrates of CYP2C9 enzyme we took one drug per cluster when available from commercially available libraries and for the largest clusters 3 and 24 we selected two and three diverse drugs, respectively. To the best of our knowledge, these 18 compounds have not been reported in the literature or in publicly available databases as experimentally validated inhibitors of CYP2C9. Their molecular structures are shown in S6 Table.

**Fig 3:**
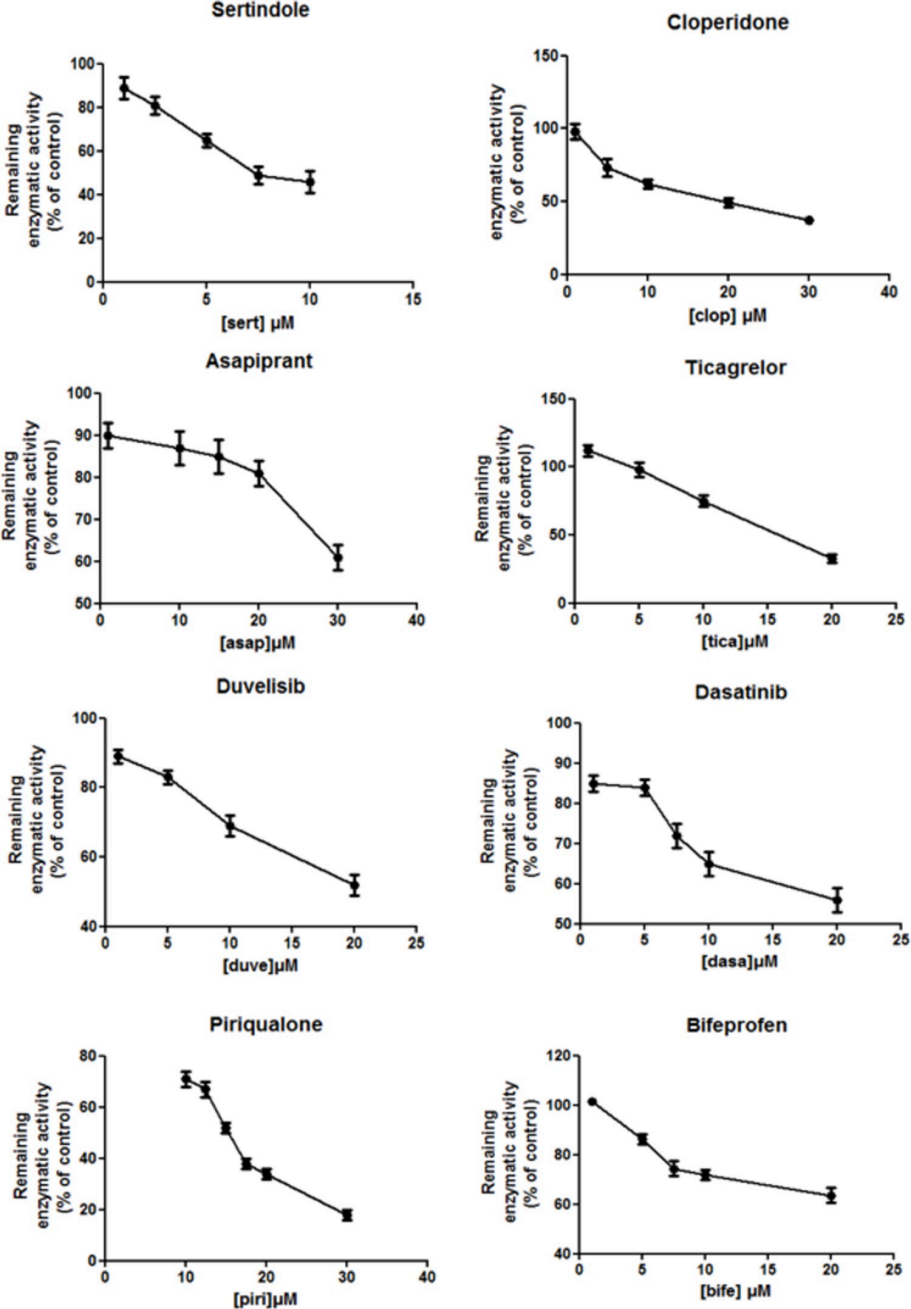
Inhibition effect of sertindole, cloperidone, asapiprant, ticagrelor, duvelisib, dasatinib, bifeprofen and piriqualone on HepG2 cells expressing human CYP2C9. The enzymatic activity with respect to the control is shown as a function of the drug concentration. HepG2 cells were treated with the drugs at the indicated concentrations for 24 h. Similar results were observed in three independent experiments. The bar graphs were obtained with GraphPadPrism v. 5.03 and represent mean ± SD of triplicate determinations.

Inhibition assays were first performed using HepG2 cells expressing high activity for the CYP2C9 enzyme. The 18 studied molecules were tested at a range of concentrations to assess the dose-dependent inhibition effect reflecting competitive inhibition mechanism towards the catalytic site of CYP2C9. The data indicated that 12 out of the 18 molecules were associated with the CYP2C9 inhibition effect, and a concentration-dependent inhibition was observed with 9 of these compounds. These results strongly suggest a competitive mechanism for enzyme inhibition and support the hypothesis that these drugs are potential inhibitors and/or substrates of CYP2C9 (Fig 3 and Table 5). The type of inhibition for the three remaining compounds (Pf-562271, ciltoprazine, tarafenacine) was not determined. However, subsequent analyses for tarafenacin on supersomes (see below) revealed that this compound is a substrate of CYP2C9 with a weak inhibition effect.

**Table 5:** *In vitro* inhibition assays of 18 tested compounds.

Drug name	Cluster	Inhibition of CYP2C9 activity in HepG2 cells* (yes/no)	Dose- dependent inhibition (yes/no)	Increased cytotoxicity in HepG2 cells expressing CYP2C9 (min [c]; % living cells)**	IC50 values determined with CYP2C9 supersomes (μM)
Abemaciclib	1	no	no	yes (5μM; 30%)	>100
Mizolastine	2	no	no	yes (50 μM; 60%)	
Sertindole	3	yes	yes	yes (10 μM; 60%)	40
Cloperidone	3	yes	yes	yes (100 μM; 40%)	17.7
Sivelestat	5	no	no	no	
Asapiprant	6	yes	yes	no	46
Pf-562271	7	yes	no	yes (10 μM; 65%)	
Ciltoprazine	8	yes	no	yes (75 μM; 0%)***	
Vatalanib	10	yes	yes	yes (20 μM; 50%)	0.067
Entinostat	11	no	no	no	
Azd3514	12	no	no	yes (20 μM; 60%)	
Muraglitazar	14	no	no	no	
Bifeprofen	16	yes	yes	yes (75 μM; 50%)	
Tarafenacin	22	yes	no	yes (50 μM; 0%)	>100
Ticagrelor	23	yes	yes	no	11.8
Duvelisib	24	yes	yes	no	52
Dasatinib	24	yes	yes	yes (20 μM; 45%)	85
Piriqualone	24	yes	yes	yes (75 μM; 0%)***	10.9

* For each compound, the inhibition tests were performed over a range of concentrations selected such that the highest concentration yielded a cell viability (based on MTS assay) greater than 80%.

** min [c]: minimal concentrations that increase the cytotoxicity (> threshold fixed at a cell viability of 80%) and percentage (%) of living HepG2 cells expressing CYP2C9 after incubation with the “min [c]” of the compound. At the same concentration, no cytotoxic effect (% living cells > 80%) was observed in the wild-type HepG2 cells incubated with the compound.

*** For ciltoprazine and piriqualone, the cytotoxicity was also observed in wt HepG2 cells at high concentrations, but the % of living cells with all concentrations (including 100 μM) was maintained at > 50%.

Interestingly, we also observed increased cytotoxicity for 12 out of the 18 compounds after incubation with HepG2 cells harboring CYP2C9 activity. These results are consistent with the cytotoxicity of metabolites produced by CYP2C9 and reinforce the hypothesis of an interaction with the enzyme. Finally, only 3 of the 18 studied compounds (sivelestat, entinostat, and muraglitazar) did not show any effect (inhibition of CYP2C9 activity and/or cytotoxicity on HepG2 cells expressing CYP2C9) over the range of concentrations used in our *in vitro* validation assays.

The inhibition assays with CYP2C9 supersomes for the estimation of IC50 values (assessment of enzyme inhibition apart from any cytotoxicity effect) were performed with 10 selected molecules. They were chosen for additional analyses since 8 of them showed dose-dependent inhibition (sertindole, cloperidone, asapiprant, vatalanib, ticagrelor, duvelisib, dasatinib, and piriqualone) and 3 of them exhibited strong cytotoxicity after incubation with HepG2 cells with CYP2C9 activity (abemaciclib, vatalanib, and tarafenacin). The inhibition data obtained using the CYP2C9 supersomes (Table 5 and S5 Fig) agreed with the results observed *in vitro* for the ten tested molecules with HepG2 cells. The analysis of abemaciclib revealed no direct inhibition of CYP2C9 in supersomes, similarly to the results obtained with HepG2 cells. These results showed that the two approaches (HepG2 cell lines expressing CYP2C9 and CYP2C9 supersomes) resulted in similar interpretations for the studied compounds.

**Fig 4:**
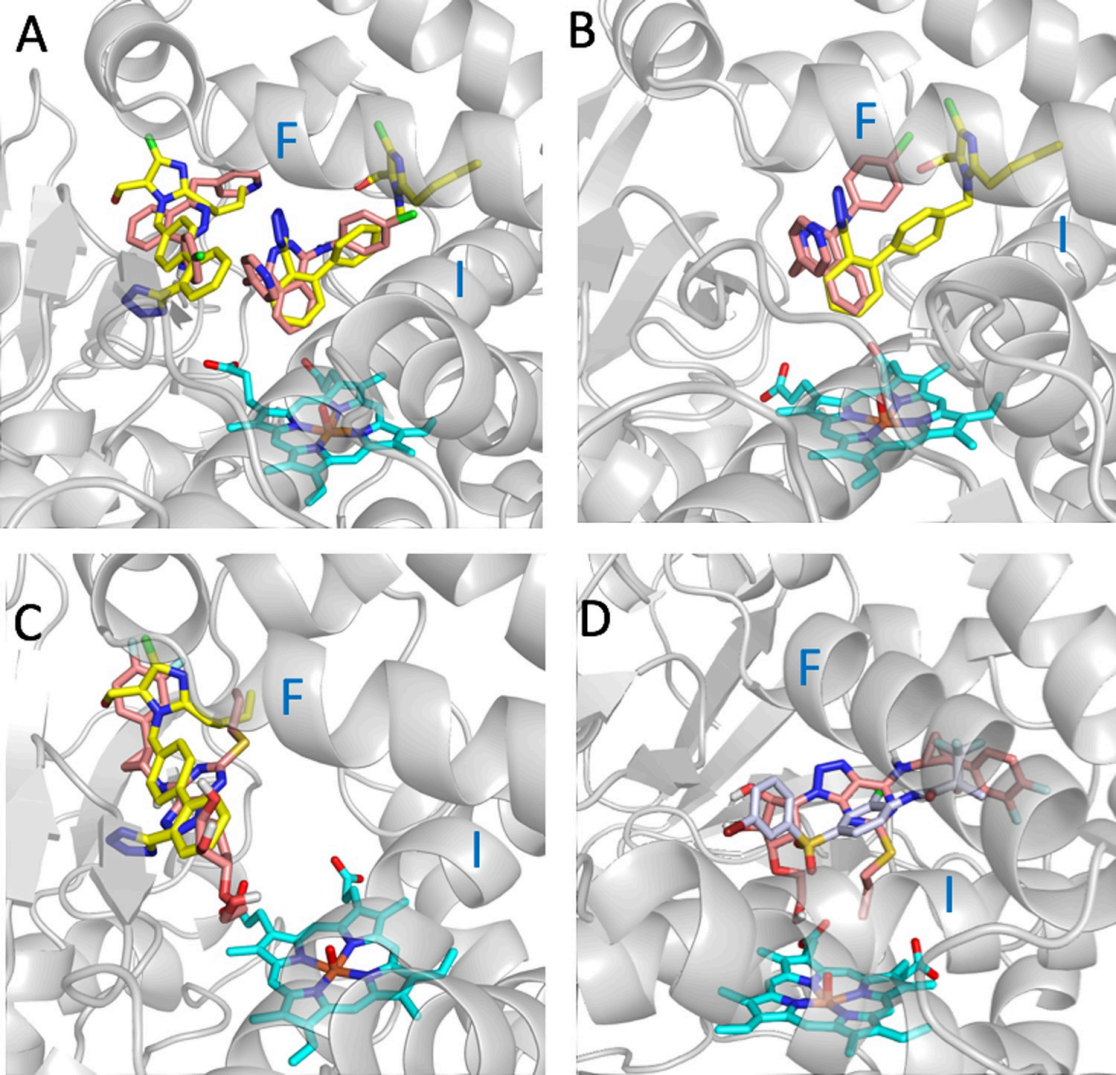
Docking conformations of vatalanib and ticagrelor in the binding pocket of CYP2C9. (A) Two poses of vatalanib (in salmon) docked into the crystal structure of CYP2C9 (PDB ID 5XXI) and the two co-crystallized molecules of losartan (PDB ID 5XXI) (in yellow). (B) The best pose of vatalanib (in salmon) docked into the MD5 structure of CYP2C9 and the superposed co-crystallized structure of losartan of the PDB ID 5XXI (in yellow). (C) The best pose of ticagrelor (in salmon) docked into the crystal structure of CYP2C9 PDB ID 5XXI and one of the two co-crystallized molecules of losartan (PDB ID 5XXI) (in yellow). (D) The best pose of ticagrelor (in salmon) docked into the MD4 structure of CYP2C9 and the superposed co-crystallized structure of the CYP2C9 inhibitor 2QJ (PDB ID 4NZ2) (in gray). Helices F and I of CYP2C9 are noted. The MD4 and MD5 structures correspond to CYP2C9 conformations generated from MD simulations of CYP2C9 bound to losartan and apo CYP2C9, respectively.

Our inhibition results showed that relatively “small” or “big” drugs can inhibit the CYP2C9 activity. The large binding pocket of CYP2C9 is composed of two smaller cavities: the so called “warfarin-binding site” (PDB ID 1OG5) [50] and the catalytic site cavity close to the heme cofactor. Interestingly, losartan can bind simultaneously in both cavities as observed in the crystal structure of CYP2C9 with co-crystallized losartan (PDB ID 5XXI). The predicted binding positions of the drugs vatalanib and ticagrelor, which have different sizes and strongly inhibit CYP2C9, are shown in Fig 4. The best poses were selected based on the predicted interaction energies of the three best scored poses. The drug poses on the left correspond to the warfarin-binding site, and those on the right correspond to the catalytic site. The docking results indicate that the two drugs can bind in the catalytic site (Fig 4A, 4B and 4D) or in the warfarin-binding site (Fig 4A and 4C). The two docking poses shown in Fig 4A suggest that it might be possible two vatalanib molecules to be simultaneously accommodated in the two cavities as is the case of losartan. The bulkier ticagrelor was predicted to be accommodated in the active site only in a MD structure with a more open binding pocket (Fig 4D). These results underline the importance of considering the flexibility of CYP2C9 for proper inhibition prediction and ligand interactions interpretation.

### Characterization of CYP2C9-produced metabolites

**Fig 5:**
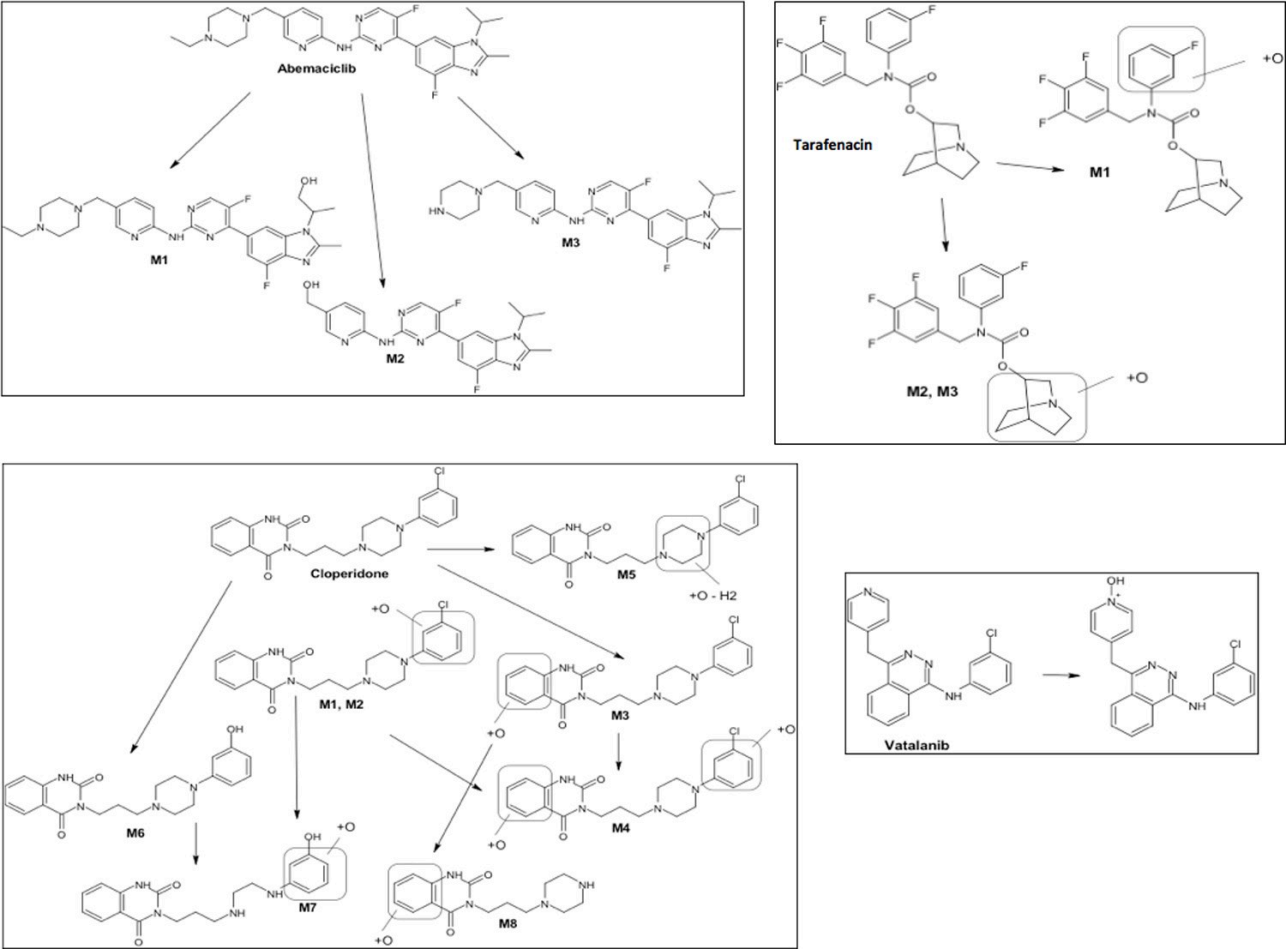
Metabolism assays using recombinant CYP2C9 supersomes for the identification of CYP2C9-produced metabolites. The suggested metabolite structures for abemaciclib, tarafenacin, cloperidone and vatalanib are shown.

We conducted metabolism assays with four molecules (abemaciclib, cloperidone, vatalanib and tarafenacin), which were selected based on their cytotoxicity after incubation with HepG2 cells expressing CYP2C9. These assays allowed the identification of the metabolites specifically produced by the CYP2C9 enzyme (Figs 5 and S6). The metabolism assays after incubation of the four candidate substrates/inhibitors with CYP2C9 supersomes allowed the characterization of specific metabolites and suggest their chemical structures, and these findings support the hypothesis of biotransformation by the CYP2C9 enzyme. Interestingly, vatalanib and cloperidone were found to strongly inhibit and to be metabolized by CYP2C9, confirming the complexity of the mechanisms. The inhibition/metabolism of CYPs can correspond to a competitive inhibition in the active site, a modification of the substrate or metabolite flux between the active site and outside of the enzyme or inhibition by a drug itself or its metabolites (time-dependent inhibition) [7]. Abemaciclib and tarafenacin showed high cytotoxicity after incubation with HepG2 cells expressing CYP2C9. They were metabolized by CYP2C9, which indicated that toxic metabolites were produced. No any inhibition effect was observed for abemaciclib. Tarafenacin showed a weak inhibition effect as discussed above. Vatalanib and cloperidone also showed increased cytotoxicity on HepG2 cells expressing CYP2C9 while strongly inhibited CYP2C9.

We then analyzed the presence of potentially toxic groups in the four drugs and their detected metabolites (Fig 5). Abemaciclib and its three metabolites M1, M2, and M3 contain a halopyrimidine group, which is an electrophilic functional toxicophore commonly known to be protein-reactive by covalent binding [51]. Although CYP3A is known as the enzyme responsible for the majority of the CYP-mediated metabolism of abemaciclib and its metabolites [52], other recent studies suggested that abemaciclib does not have a clinically meaningful effect on pharmacokinetics of CYP1A2, CYP2C9, CYP2D6, and CYP3A4 substrates in patients with cancer [53]. Oxidative metabolism of the phenol functionality present in all identified metabolites of cloperidone (M1, M2, M3, M4, M6, M7, M8) but M5 can lead to the formation of reactive catechol/quinone/quinone-imine intermediates [33,54]. We did not find common toxic groups in the CYP2C9 produced metabolites of tarafenacin and vatalanib. Extensive hepatic metabolism mostly CYP3A4-mediated is known for vatalanib [55]. Interestingly, a recent study on oxidation of the anticancer drugs sunitinib and pazopanib using a chemical catalytic system able to mimic CYP type oxidation allowed to identify reactive/toxic metabolites of these two drugs [56]. Low amount of aldehyde derivatives was detected, those metabolites have not been previously identified by NMR spectroscopy. Such derivative aldehydes have been expected to quickly react with amines and can be considered as potentially toxic. As suggested in [56], such carboxaldehyde metabolites can escape detection in metabolite studies due to their high reactivity, but they could be intermediates explaining the hepatotoxicity of sunitinib and pazopanib. One may speculate that the chemical similarities present for some substructures of these two drugs and abemaciclib and vatalanib suggest possible production of highly reactive aldehyde intermediates that were not detected by our metabolite assays.

## Conclusion

We developed and validated a new ML approach for the prediction of CYP2C9 inhibition. We built predictive models by combining an original selection of physicochemical descriptors of CYP2C9 inhibitors, CYP2C9 protein structure and dynamics knowledge, and machine learning SVM and RF modeling. The validation on PubChem and ChEMBL data demonstrated that our models successfully predicted CYP2C9 inhibitors with an accuracy, sensitivity and specificity of approximately 80%. The application of this approach allowed to propose 18 drugs for *in vitro* validation of CYP2C9 inhibition. We provide here the first identification of the drugs vatalanib, piriqualone, ticagrelor and cloperidone as strong inhibitors of CYP2C9 with IC values <18 μM and sertindole, asapiprant, duvelisib and dasatinib as moderate inhibitors with IC50 values between 40 and 85 μM. Vatalanib was identified as the strongest inhibitor with an IC50 value of 0.067 μM. Metabolism assays allowed the characterization of specific metabolites of abemaciclib, cloperidone, vatalanib and tarafenacin produced by CYP2C9. The obtained results demonstrate that such a strategy could improve the prediction of drug-drug interactions in clinical practice and could be utilized to prioritize drug candidates in drug discovery pipelines.

## Methods

### Training and external test data sets preparation

We used the PubChem BioAssay datasets of CYP2C9 AID 883 and 1851 containing data of CYP2C9 inhibition for 27463 chemical compounds. In addition, we collected 5114 compounds from ChEMBL tested for CYP2C9 (the ChEMBL IDs are given in S1 Text). We retained the 8851 most active inhibitors from PubChem and ChEMBL data with AC50 (or IC50) values ≤ 10 μM (AC50, “activity concentration 50” refers to the concentration that is required to elicit the half-maximal effect), and 6056 non-inhibitors from PubChem showing less than 10% inhibition at a concentration of 50 μM. This collection was filtered for duplicates using the web server FAF-Drugs4 [33] and an in-house-developed “soft” drug-like filter for physicochemical properties (see S1 Text) without removing toxic/reactive/PAINS (Pan Assay Interference) compounds since toxic or PAINS compounds could also be CYP inhibitors. The 3D compound structures were generated using the freely available web server Frog2 [57], and the compounds were protonated at pH 7.4 using the major macrospecies option of the ChemAxon calculator plugins (www.chemaxon.com). Gasteiger atom charges were added using the AutoDockTools package [58]. Structural clustering was performed using OSIRIS DataWarrior software [49] with the FragFp Structure descriptor and a similarity cutoff of 0.85. Only the cluster centroids were taken resulting in the final filtered and diverse dataset containing 4840 inhibitors and 3301 non-inhibitors. The training set was built by randomly chosen 80% of both active and inactive compounds of the final dataset. The remaining 20% of the molecules were used as external test set.

### Ensemble docking

Two X-ray CYP2C9 structures were obtained from the Protein Data Bank (PDB): 5XXI co-crystallized with losartan [34] and 1R9O [35] co-crystallized with flurbiprofen. The MD structures were generated from previously performed MD simulations [30]. The pKa values of the titratable groups of CYP2C9 were calculated with the FDPB approach using the PCE web server [59]. The charges were assigned using the AutoDockTools package [58]. The heme was kept in the intermediate Cpd I state [60] since our preliminary tests showed similar docking scores calculated using Cpd I or ferric heme. We performed virtual screening and docking of the dataset compounds using the well-established free software AutoDock Vina [36], which employs gradient-based conformational docking and an empirical scoring function predicting the protein-ligand binding energy in kcal/mol. The grid resolution was set to 1 Å, the maximum number of output binding modes was fixed to 10, and the exhaustiveness level was set to 8. The grid included the whole binding pocket of cytochrome CYP2C9. The grid center coordinates used were 8.208, 32.219, -1.923, and the size of the search space was set to 25 Å×25 Å×25 Å according to PDB ID 1R9O. The best docking score for each protein conformation was retained. The docking performance of Autodock Vina for CYP2C9 was checked by preliminary docking of flurbiprofen into the structure of CYP2C9 co-crystallized with flurbiprofen (PDB ID 1R9O).

### Machine learning classification modeling

#### Random forest

RF classification [61] was performed using the Random Forest R library [62] of the statistical software package R. Multiple decision trees were built with bootstrap samples from the training data. To introduce diversity between the trees of the RF, a small subset of descriptors was randomly selected to make decisions at each node of each tree. The classification was obtained by taking the results of all the trees through a majority vote. To find the optimal size of the forest (*ntree* is the number of trees) and the number of descriptors (*mtry* is the number of selected descriptors) for each model based on different numbers of descriptors, we ran RF calculations scanning over the *ntree* (25–500) and *mtry* (5–13) parameters (see SI for details). The maximum value of *mtry* was set to 13 according to the widely accepted concept that the value of *mtry* should be equal to *√p*, where *p* is the total number of variables [63]. For each model, we selected the combinations of *ntree* and *mtry* parameters that yield the best internal accuracy while retaining the lowest acceptable *ntree* (see S3 Table). A ten-fold cross-validation procedure that was repeated five times.

#### Support vector machine

SVMs are based on the minimization principle from statistical learning theory and place data into a hyperspace through a kernel function for its separation into datasets for classification or regression modeling [64]. For the nonlinearly separable cases, the kernel function allows SVM to transfer the data points into a higher-dimensional space where linear separation is possible. To build the classification models, we used the SVM algorithms implemented in the R package with the Caret library [65]. The descriptors were centered around a mean of 0 and scaled to a variance equal to 1. We selected the **radial basis function** kernel (SVM-Rad). The cost parameter was optimized in the range of 2^−2^–2^7^ through a ten-fold cross-validation procedure that was repeated five times. The best combination of the hyperparameter cost and scaling function sigma is shown in S3 Table.

## Experimental materials and methods

### Chemical and reagents

The chemical compounds included in the inhibition assay screening are shown in S6 Table. The compounds used for *in vitro* testing were dissolved in DMSO when received at the laboratory. Depending on the molecule and its solubility in DMSO, aliquots (50 μl) of stock solutions with concentrations ranging from 8 mM to 25 mM were prepared. The stock solutions were stored at −20°C until use on the experiment day.

### Cell culture and plasmids

HepG2 cells (ATCC-HB-8065; Lot/Batch: 70007613) were maintained at 37°C and 5% CO_2_ in Minimum Essential Medium (Gibco, Life Technologies) containing 10% fetal bovine serum (HyClone GE Healthcare) and supplemented with penicillin (200 UI/mL), streptomycin (50 μg/mL), L-glutamine (0.3 mg/mL), and sodium pyruvate (1 mM). The cells were tested for mycoplasmas (Mycoplasma PCR Detection Kit, Merck). Cloning, *in vitro* mutagenesis and sequencing of CYP2C9 cDNA (sequence reference: NM_000771) were performed by Eurofins Genomics (Germany).

### Selection of stable HepG2 clones expressing high CYP2C9 enzyme activity

HepG2 cells were infected with 2.5x10^9^ TU/mL lentivirus (pLentiIII-EF1alpha) containing the CYP2C9 wild-type sequence produced by the « Plateforme Vecteurs viraux et Transfert de gènes » (University of Paris). After transducing the lentiviral construct into the HepG2 cell line, a range of multiplicities of infection (MOIs) of 10, 30, and 50 (2, 6 and 10 μL of lentiviral particles per 5 x 10^5^ cells) was used to determine the optimal transduction efficiency, and the highest activity of the CYP2C9 enzyme measured by P450-Glo CYP2C9 Assays (Promega, France) was obtained with an MOI of 30. Recombinant clones were selected in the presence of 2 μg.mL^-1^ puromycin and then expanded to assay for CYP2C9 enzymatic activity.

### Cytotoxicity assays and selection of the concentration ranges of the study chemical compounds

For the assessment of cell viability, “wild type” (wt) HepG2 cells (without CYP2C9) and HepG2 cells expressing the CYP2C9 enzyme were incubated with serial dilutions of selected molecules (at concentrations from 10 nM to 100 μM). Briefly, HepG2 cells were incubated in 96-well plates (2.5x 10^4^ cells/mL), and the number of living cells was determined by the MTS assay according to the protocol recommended by the manufacturer (Promega, France). For each compound, the inhibition test was performed over a range of concentrations selected such that the highest concentration yielded a cell viability greater than 80% to minimize any biased interpretation of the results due to direct cytotoxicity of the parent compounds or their potential metabolites produced by CYP2C9.

### CYP2C9 enzymatic activity and inhibition assays

#### P450-Glo activity

The P450-Glo CYP2C9 Assays with Luciferin-H kit (Promega, France) was used to measure cytochrome P450 2C9 activity. Luciferin H is proluciferin, a derivative of beetle luciferin. This derivative is converted by the CYP2C9 enzyme to luciferin products. D-luciferin is formed and detected via a second reaction with the Luciferin Detection Reagent. The amount of light produced in the second reaction is directly proportional to CYP activity. Luciferase-free water was added in the wells reserved for background luminescence. After 4 h of incubation, the enzyme reaction was stopped by the addition of 50 μl of Luciferin Detection Reagent, which also contains the esterase needed for generation of the luminescence signal. In each plate, a standard range of beetle luciferin (from 8 nM to 224 nM) was included. The plate was finally incubated at room temperature in the dark for 30 min to stabilize the luminescence signal, which was measured using an EnSpire plate reader (Perkin Elmer).

#### Kinetics of inhibition tests with HepG2 cells expressing CYP2C9

Concentration-dependent CYP2C9 inhibition assays were performed by preparing a five-step dilution series from 0.5 (min) to 50 μM (max) of selected compounds according to the viability tests and by transferring 12.5 μl of each dilution into the assay plate. CYP2C9 activity was determined (as described above) and compared to that of untreated controls incubated with DMSO buffer alone. Each compound dilution was tested in triplicate in the inhibition assays, and the experiments were independently repeated on three different days. The data were analyzed with GraphPad Prism Version 5.03 software.

#### Quantification of the luminogenic signal

The raw data obtained from the EnSpire plate reader were processed by calculating the total luminescence (% of untreated control) using the following formula: raw data − (background luminescence) * 100/mean of untreated control. All the values were normalized to the amount of proteins extracted from fresh cells with Pierce RIPA buffer (Thermo Scientific, France). CYP2C9 enzyme activity is expressed in pmol D-luciferin.mg^-1^ of protein.min^-1^.

### Inhibition of CYP2C9 on recombinant supersomes and calculation of IC50

A CYP2C9-specific substrate (diclofenac) and recombinant supersomes were incubated with and without the study compounds (according to the protocol optimized by the Service Provider Admescope (Finland)). The concentrations of the studied compounds were 0.3, 1, 3, 10, 20, 30, 50, 60, 70, 90 and 100 μM. The substrate of CYP2C9 used in the inhibition test was diclofenac at 5 μM, and the specific metabolite produced was 4-hydroxylated diclofenac. The selective CYP2C9 inhibitor sulfaphenazole was used as a control in the reactions (IC50 value estimated between 0.2 and 0.4 μM). The time points used in the analysis were 0, 10, 20, 40, and 60 min. The enzymatic reactions were started after 6 min of preincubation by adding NADPH and terminated after 15 min by adding ice-cold acetonitrile. The supernatant was collected and centrifuged for analyses. The samples were analyzed by LC/MS-MS to determine the level of metabolites in the absence and presence of a candidate molecule. Spiked standard samples were not used, but quantification based on relative peak areas was performed (solvent control = 100%). The disappearance was evaluated as relative LC/MS peak areas, and the 0-min time points were marked as 100%. The disappearance rate was used to calculate the half-life and *in vitro* clearance. For details of the method, see S2 Text.

### Identification of the metabolites of the studied compounds produced by CYP2C9

The metabolites were identified according to the protocol developed by Admescope (Finland). Briefly, the studied compounds were incubated with recombinant CYP2C9 supersomes. The selective CYP2C9 substrate diclofenac was used as a control at 1 μM. The time points used in the analysis were 0, 10, 20, 40, and 60 min, and the collected samples were analyzed by UPLC/HR-MS to monitor substrate depletion. The collected samples were stored at -20°C until thawed, centrifuged and analyzed for substrate depletion by UPLC/HR-MS, and the analytical method was optimized using the parent compounds for fit-for-purpose chromatographic properties (peak shape and retention) and mass spectrometric ionization.

## Supporting information

S1 Text Details of machine learning methods. (PDF)

S2 Text Details of analytical method for inhibition tests using CYP2C9 supersomes. (PDF)

S1 Fig Chemical space of the training and external test sets. (PDF)

S2 Fig MD and crystal CYP2C9 structures used for ML modeling. (PDF)

S3 Fig Autodock Vina scores of the training set’ inhibitors and non-inhibitors calculated on seven different CYP2C9 conformations. (PDF)

S4 Fig Mean importance of 177 descriptors used to train ML models. (PDF)

S5 Fig Kinetics of inhibition observed for the studied compounds with CYP2C9 supersomes. (PDF)

S6 Fig Details of metabolism assays for the identification of CYP2C9-produced metabolites. (PDF)

S1 Table Mean importance of the best 36 physicochemical descriptors and 7 IEs used to train ML models. (PDF)

S2 Table Performances of preliminary RF models with MOE descriptors on the training set. (PDF)

S3 Table Parameters of the optimized best RF and SVM models. (PDF)

S4 Table Performances of the optimized RF and SVM models with MOE descriptors on the external validation set. (PDF)

S5 Table Comparison of datasets used and performances of models as reported in the literature. (PDF)

S6 Table List of the experimentally tested drugs. (PDF)
