# Supplementary material for: Machine learning-driven identification of drugs inhibiting cytochrome P450 2C9
Source: PLoS Comput Biol. 2022 Jan 26;18(1):e1009820. doi: 10.1371/journal.pcbi.1009820 (PMC8820617; doi:10.1371/journal.pcbi.1009820)
Supplement: S1 Text — (PDF) [file pcbi.1009820.s001.pdf]

Details of machine learning methods

ChEMBL assays IDs used for training and test sets:

CHEMBL1010069, CHEMBL1013136, CHEMBL1030555, CHEMBL1034783, CHEMBL1042607, CHEMBL1066806, CHEMBL1072025, CHEMBL1073470, CHEMBL1103798, CHEMBL1103804, CHEMBL1104622, CHEMBL1117614, CHEMBL1167157, CHEMBL1167163, CHEMBL1219167, CHEMBL1219236, CHEMBL1219458, CHEMBL1246349, CHEMBL1259939, CHEMBL1274769, CHEMBL1293079, CHEMBL1646003, CHEMBL1646061, CHEMBL1647680, CHEMBL1768199, CHEMBL1768380, CHEMBL1768381, CHEMBL1780619, CHEMBL1816327, CHEMBL1827183, CHEMBL1827531, CHEMBL1833284, CHEMBL1837191, CHEMBL1840132, CHEMBL1920118, CHEMBL1924559, CHEMBL1925277, CHEMBL1942058, CHEMBL1958502, CHEMBL2019954, CHEMBL2025322, CHEMBL2026797, CHEMBL2033875, CHEMBL2038382, CHEMBL2045911, CHEMBL2050711, CHEMBL2161926, CHEMBL2162551, CHEMBL2183961, CHEMBL2317466, CHEMBL2319720, CHEMBL2320894, CHEMBL2327408, CHEMBL2327764, CHEMBL2340395, CHEMBL2341174, CHEMBL2343699, CHEMBL2346258, CHEMBL2350016, CHEMBL2350100, CHEMBL2384500, CHEMBL2384506, CHEMBL2432690, CHEMBL2432691, CHEMBL2445671, CHEMBL3095185, CHEMBL3095759, CHEMBL3128584, CHEMBL3266924, CHEMBL3268321, CHEMBL3268923, CHEMBL3294170, CHEMBL3364721, CHEMBL3366947, CHEMBL3372275, CHEMBL3373958, CHEMBL3375953, CHEMBL3382240, CHEMBL3383256, CHEMBL3389065, CHEMBL3390677, CHEMBL3404109, CHEMBL3404692, CHEMBL3411304, CHEMBL3414225, CHEMBL3420147, CHEMBL3508953, CHEMBL3508954, CHEMBL3508955, CHEMBL3535106, CHEMBL3599702, CHEMBL3611646, CHEMBL3611884, CHEMBL3616248, CHEMBL914447, CHEMBL919483, CHEMBL927600, CHEMBL927601, CHEMBL927774, CHEMBL930918, CHEMBL933754, CHEMBL942002, CHEMBL954327, CHEMBL961565, CHEMBL971583, CHEMBL971594

Soft drug-like filter: An in-house-developed "soft" drug-like filter for physicochemical properties implemented in the web server FAF-Drugs4 was used to filter the collected datasets of inhibitors and non-inhibitors of CYP2C9 based on the following rules: molecular weight ≥ 1000 Da, number of H-bond donors ≤ 8, number of H-bond acceptors ≤ 12, rotatable bonds ≤ 20, logP (XlogP3) and logD between -7 and 10, and heteroatoms ≤ 15. Toxic and PAINS groups were not removed from the compounds data sets.

Random Forrest scanning parameters: The following values of *ntree* were used: 25, 75, 125, 175, 200, 225, 250, 275, 300, 400, and 500. The following values of *mtry* were used: 5, 7, 9, 11, and 13 parameters.

Assessment of the quality of the models:

To assess the predictive ability of the models, the following properties were calculated:

- Sensitivity is the fraction of true positives among all positively classified instances (true positive rate) and is calculated as follows: Sensitivity = TP/(TP+FN).
- Specificity is the true negative rate and is calculated as Specificity = TN/(TN+FP)
- Accuracy is the proportion of true results, either true positive or true negative, and is calculated as follows:

$$Accuracy=(TP+TN)/(TP+TN+FP+FN)$$

- Matthew's correlation coefficient (MCC) was calculated to measure the quality of binary classification as follows:

$$MCC = (TP*TN - FP*FN)/((TP+FP) * (TP+FN) * (TN+FP) * (TN*FN))$$

"TP" and "TN" are the true positive and true negative instances, respectively, and "FP" and "FN" are the false positive and false negative instances, respectively. A value of 0 indicates random and 100 perfect predictions).

Preparation of the 4480 drugs for screening

Four compound databases were used to generate the library of drugs used in this study to screen for inhibitors of CYP2C9: the "drug" subset of the ChEMBL database [1], the "approved" subset of DrugBank [2], the DrugCentral database [3], and the "approved" SuperDrug2 database [4]. FAF-Drugs4 [5] was used to remove isotopes, inorganics, mixtures, salts and duplicates. Additional comments about the preparation of these molecules were discussed by Largarde et al. [6]. The compounds were protonated at pH 7.4 using the major macrospecies option of the ChemAxon calculator plugins (www.chemaxon.com). CORINA Classic (Molecular Networks, www.mn-am.com) was used for 3D conformation generation as preserving the existing stereocenters. Gasteiger atom charges were added using the AutoDockTools package. We removed the compounds with missing MOE descriptors calculated as well as drugs for which experimental data regarding the inhibition of CYP2C9 were found in the literature. Finally, 4480 drugs remained for screening.

References

1. Gaulton, A., Hersey, A., Nowotka, M., Bento, A. P., Chambers, J., Mendez, D., Mutowo, P., Atkinson, F., Bellis, L. J., Cibrian-Uhalte, E., Davies, M., Dedman, N., Karlsson, A., Magarinos, M. P., Overington, J. P., Papadatos, G., Smit, I., and Leach, A. R. (2017) The ChEMBL database in 2017, *Nucleic Acids Res 45*, D945-D954.
2. Wishart, D. S., Knox, C., Guo, A. C., Shrivastava, S., Hassanali, M., Stothard, P., Chang, Z., and Woolsey, J. (2006) DrugBank: a comprehensive resource for in silico drug discovery and exploration, *Nucleic Acids Research 34*, D668-D672.
3. Ursu, O., Holmes, J., Knockel, J., Bologa, C. G., Yang, J. J., Mathias, S. L., Nelson, S. J., and Oprea, T. I. (2016) DrugCentral: online drug compendium, *Nucleic Acids Research 45*, D932-D939.
4. Siramshetty, V. B., Eckert, O. A., Gohlke, B. O., Goede, A., Chen, Q., Devarakonda, P., Preissner, S., and Preissner, R. (2018) SuperDRUG2: a one stop resource for approved/marketed drugs., *Nucleic Acids Res. 46(D1)*, D1137-D1143.
5. Lagorce, D., Bouslama, L., Becot, J., Miteva, M. A., and Villoutreix, B. O. (2017) FAF-Drugs4: free ADME-tox filtering computations for chemical biology and early stages drug discovery., *Bioinformatics 33*, 3658-3660.
6. Lagarde, N., Rey, J., Gyulkhandanyan, A., Tufféry, P., Miteva, M. A., and Villoutreix, B. O. (2018) Online structure-based screening of purchasable approved drugs and natural compounds: retrospective examples of drug repositioning on cancer targets. , *Oncotarget 9*, 32346-32361.
