# Supplementary material for: Machine learning-driven identification of drugs inhibiting cytochrome P450 2C9
Source: PLoS Comput Biol. 2022 Jan 26;18(1):e1009820. doi: 10.1371/journal.pcbi.1009820 (PMC8820617; doi:10.1371/journal.pcbi.1009820)
Supplement: S2 Text — (PDF) [file pcbi.1009820.s002.pdf]

Details of analytical method for inhibition tests using CYP2C9 supersomes and calculation of IC50

Test compound concentrations:

Test compound	Test concentrations (µM)
Abemaciclib	0.3, 1, 3, 10, 20 and 30 µM<br>10, 30, 50, 60, 70 and 80 µM
Sertindole	0.3, 1, 3, 10, 20 and 30 µM<br>10, 30, 60, 70, 90 and 100 µM
Asapiprant	0.3, 1, 3, 10, 20 and 30 µM<br>10, 30, 60, 70, 90 and 100 µM
Tarafenacin	0.3, 1, 3, 10, 20 and 30 µM<br>10, 30, 60, 70, 90 and 100 µM
Duvelisib	0.3, 1, 3, 10, 20 and 30 µM<br>10, 30, 60, 70, 90 and 100 µM
Dasatinib	0.3, 1, 3, 10, 20 and 30 µM<br>10, 30, 60, 70, 90 and 100 µM
Cloperidone	0.3, 1, 3, 10, 20 and 30 µM
Vatalanib	0.3, 1, 3, 10, 20 and 30 µM
Ticagrelor	0.3, 1, 3, 10, 20 and 30 µM
Piriqualone	0.3, 1, 3, 10, 20 and 30 µM

Substrates and concentrations: Diclofenac at 5 µM for CYP2C9

Instrumentation: Waters Acquity UPLC + Thermo TSQ Endura triple quadrupole MS
Software: XCalibur 4.0

Calculations

The IC_{50} value for the test compound was determined by fitting following equation to the data

$$A\% = \frac{Top - Bottom}{1 + 10^{(Log[I] - LogIC_{50})}} + Bottom$$

A% = percentual activity remaining
Top = the upper plateau of percentual activity (constrained to 100)
Bottom = the lower plateau of percentual activity (constrained to 0)
[I] = inhibitor concentration
IC_{50} = inhibitor concentration, where the remaining activity is 50%
Software: GraphPad prism 8.02
