# Supplementary material for: Machine learning-driven identification of drugs inhibiting cytochrome P450 2C9
Source: PLoS Comput Biol. 2022 Jan 26;18(1):e1009820. doi: 10.1371/journal.pcbi.1009820 (PMC8820617; doi:10.1371/journal.pcbi.1009820)
Supplement: S1 Fig — (PDF) [file pcbi.1009820.s003.pdf]

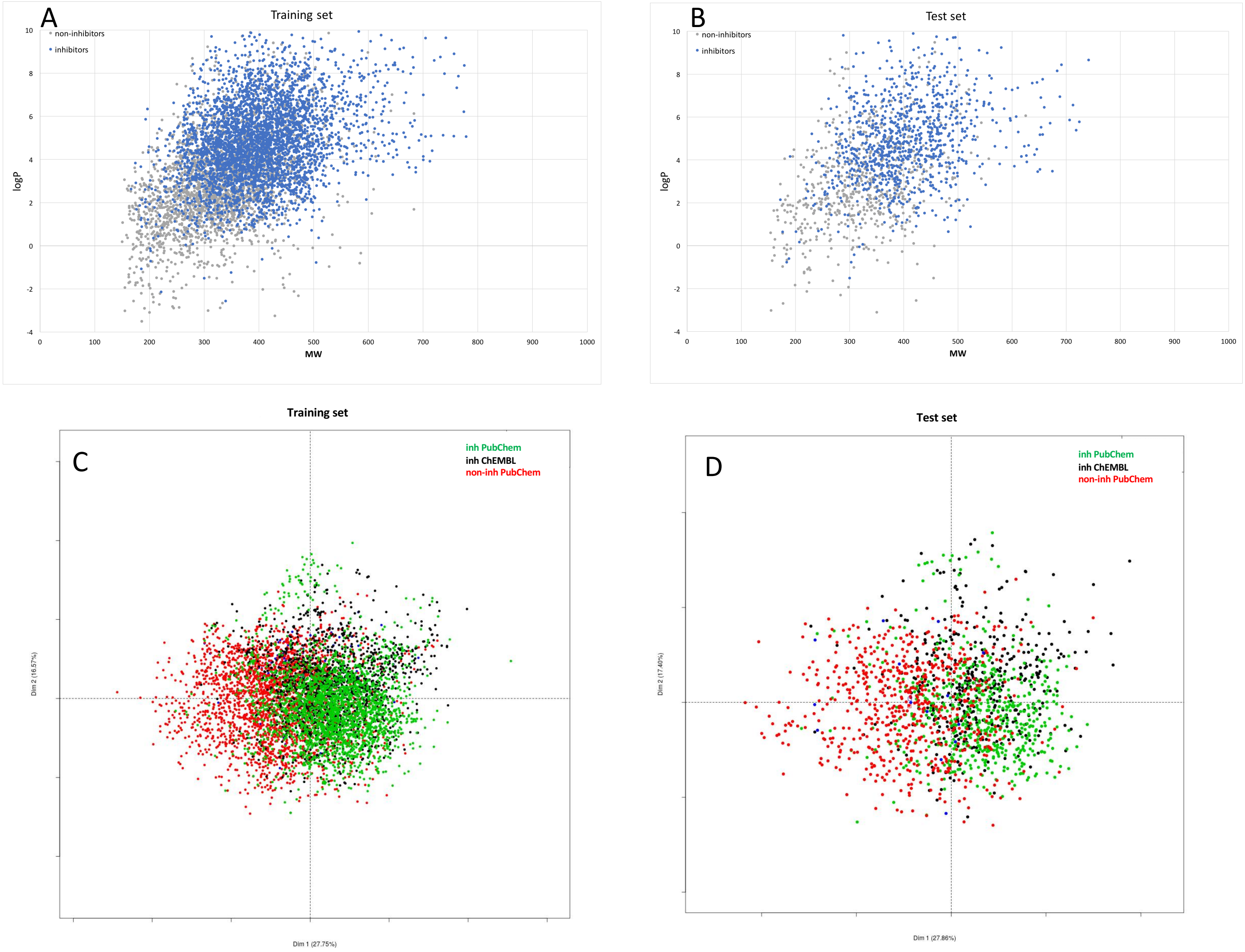

Figure S1. Chemical space of the training and external test sets. A. Comparison of logP and molecular weight (MW) values of the training set' inhibitors and non-inhibitors. B. Comparison of logP and MW values of the test set' inhibitors and non-inhibitors. C. PCA of the training set' inhibitors and non-inhibitors. D. PCA of the test set' inhibitors and non-inhibitors.
