# Supplementary material for: Machine learning-driven identification of drugs inhibiting cytochrome P450 2C9
Source: PLoS Comput Biol. 2022 Jan 26;18(1):e1009820. doi: 10.1371/journal.pcbi.1009820 (PMC8820617; doi:10.1371/journal.pcbi.1009820)
Supplement: S2 Fig — (PDF) [file pcbi.1009820.s004.pdf]

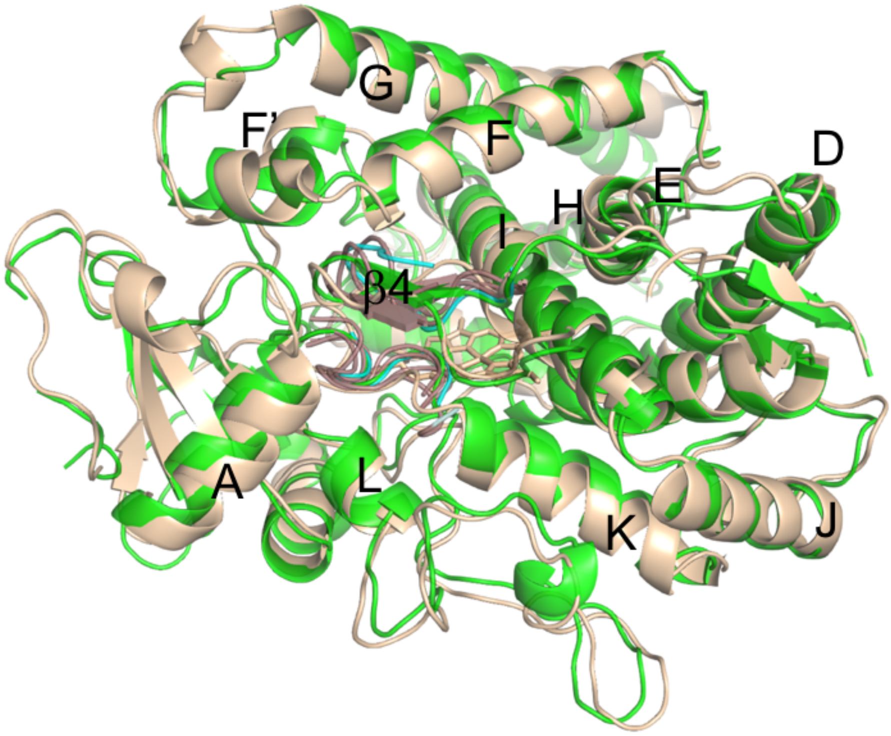

Figure S2. The seven CYP2C9 conformations chosen for the ML modeling. The X-ray structure of CYP2C9 co-crystallized with losartan (PDB ID 5XXI) is shown in green. One of the MD conformations (MD2) is shown in salmon. The heme is shown in sticks. The two loops that surround the substrate binding site are highlighted in brown for the other four MD conformations and in cyan for the X-ray structure of CYP2C9 co-crystallized with with flurbiprofen (PDB ID 1R9O). The helices and the turn β4 are noted.
