# Supplementary material for: Machine learning-driven identification of drugs inhibiting cytochrome P450 2C9
Source: PLoS Comput Biol. 2022 Jan 26;18(1):e1009820. doi: 10.1371/journal.pcbi.1009820 (PMC8820617; doi:10.1371/journal.pcbi.1009820)
Supplement: S3 Fig — (PDF) [file pcbi.1009820.s005.pdf]

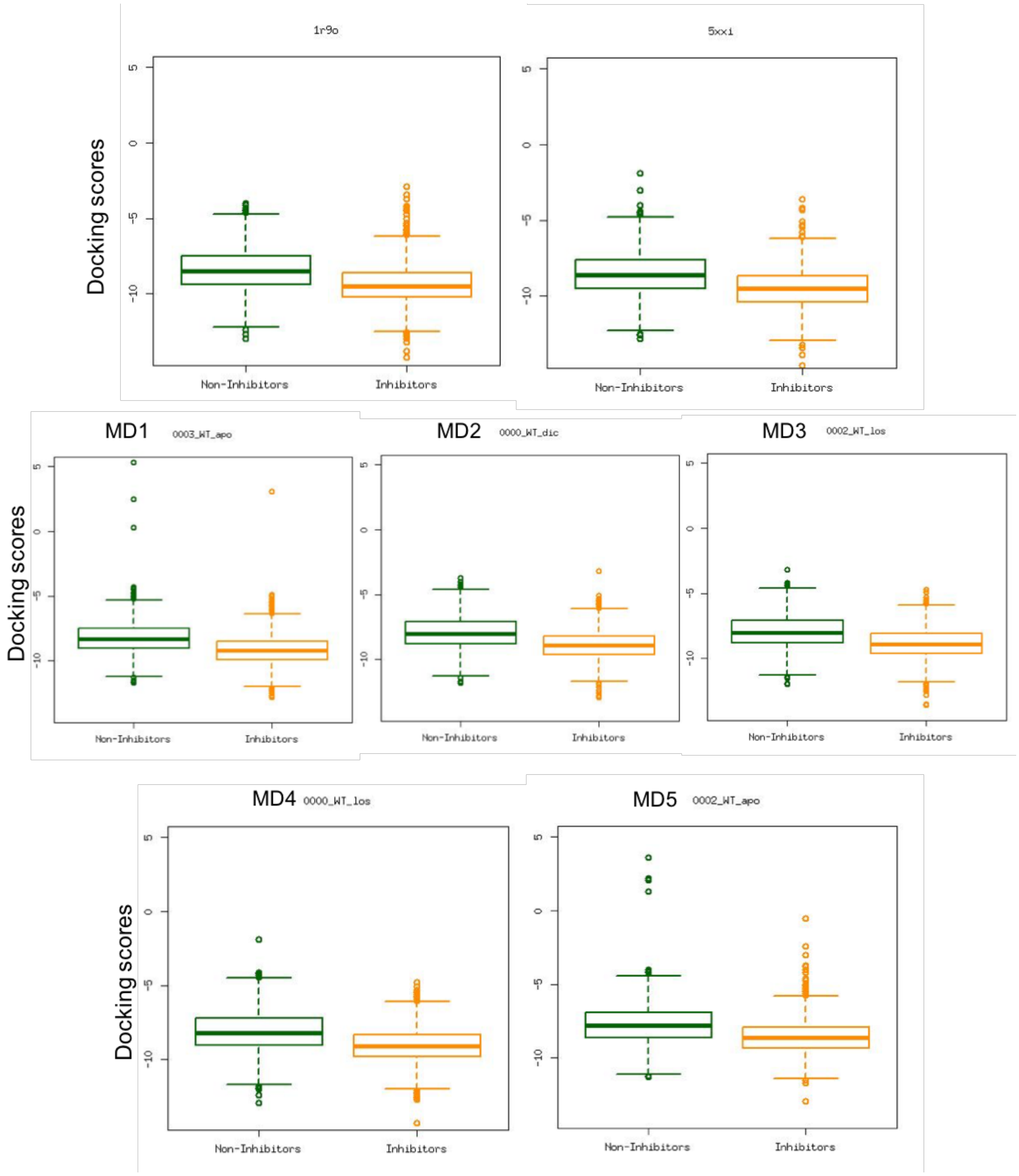

Figure S3. Autodock Vina scores of the training set' inhibitors and non-inhibitors calculated on seven different CYP2C9 conformations.
