# Supplementary material for: Machine learning-driven identification of drugs inhibiting cytochrome P450 2C9
Source: PLoS Comput Biol. 2022 Jan 26;18(1):e1009820. doi: 10.1371/journal.pcbi.1009820 (PMC8820617; doi:10.1371/journal.pcbi.1009820)
Supplement: S4 Fig — (PDF) [file pcbi.1009820.s006.pdf]

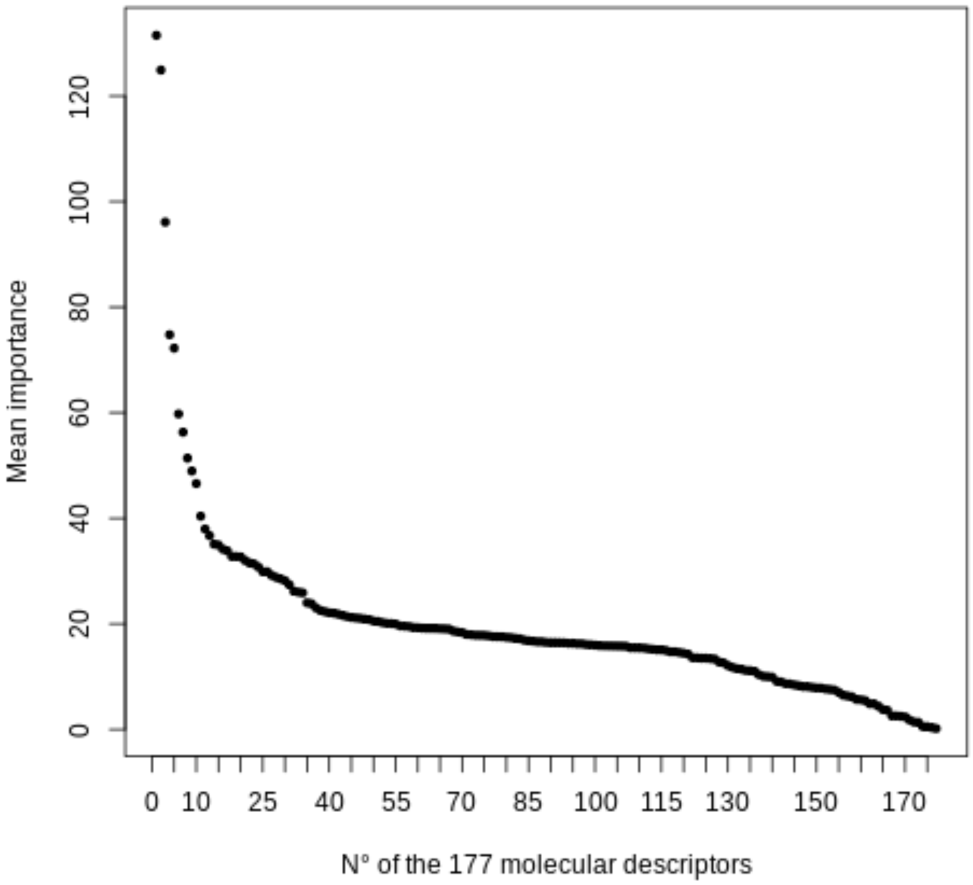

Figure S4. Mean importance inferred from the random forest modeling of the 177 molecular descriptors used to train models for CYP2C9 prediction.
