# Supplementary material for: Machine learning-driven identification of drugs inhibiting cytochrome P450 2C9
Source: PLoS Comput Biol. 2022 Jan 26;18(1):e1009820. doi: 10.1371/journal.pcbi.1009820 (PMC8820617; doi:10.1371/journal.pcbi.1009820)
Supplement: S5 Fig — (PDF) [file pcbi.1009820.s007.pdf]

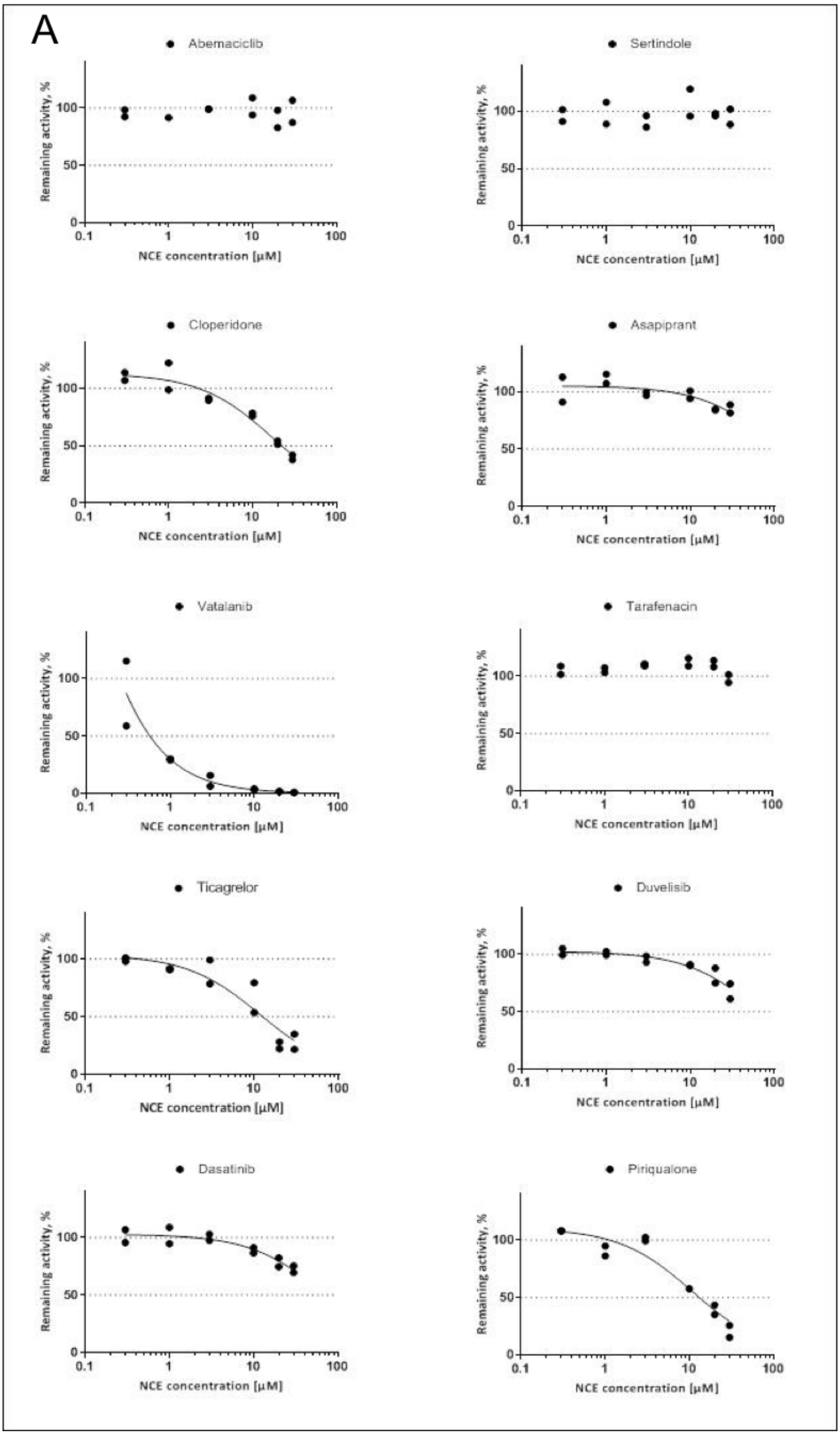
A
Abemaciclib
Sertindole
Cloperidone
Asapiprant
Vatalanib
Tarafenacin
Ticagrelor
Duvelisib
Dasatinib
Piriqualone
Remaining activity, %
NCE concentration [μM]
0
50
100
0.1
1
10
100

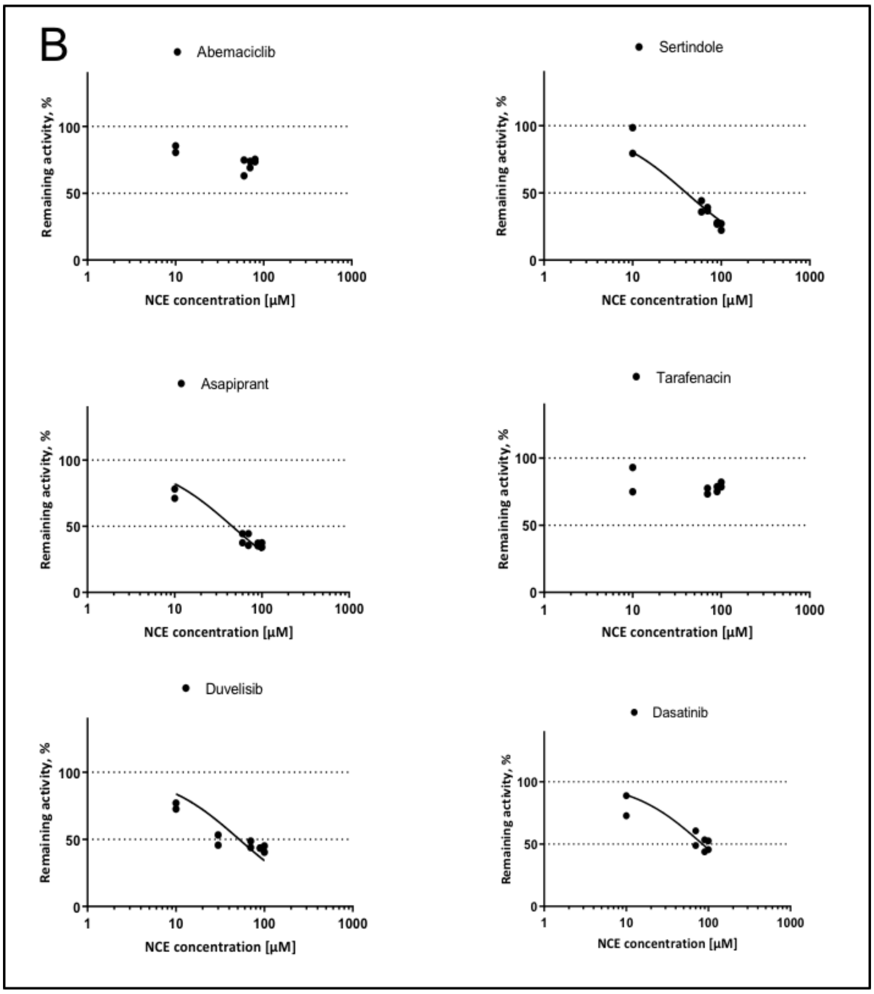

Figure S5. Kinetics of inhibition observed for the studied compounds with CYP2C9 supersomes. Two independent experiments were done. A The test concentration ranged from 0.3 to 30 μM. B The test concentration ranged from 10 to 100 μM.
