# Supplementary material for: Machine learning-driven identification of drugs inhibiting cytochrome P450 2C9
Source: PLoS Comput Biol. 2022 Jan 26;18(1):e1009820. doi: 10.1371/journal.pcbi.1009820 (PMC8820617; doi:10.1371/journal.pcbi.1009820)
Supplement: S6 Fig — (PDF) [file pcbi.1009820.s008.pdf]

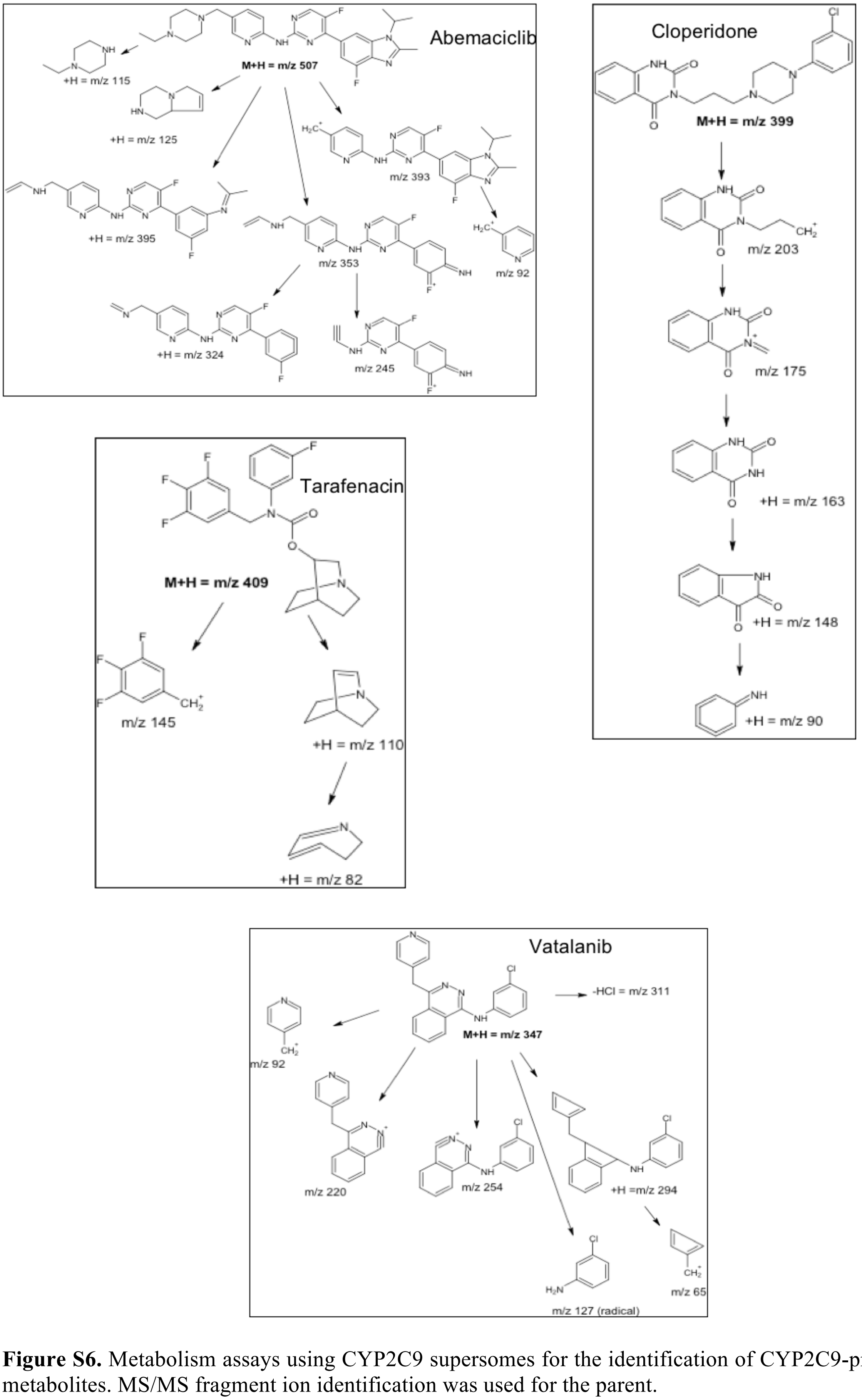

Figure S6. Metabolism assays using CYP2C9 supersomes for the identification of CYP2C9-produced metabolites. MS/MS fragment ion identification was used for the parent.
