# Supplementary material for: Machine learning-driven identification of drugs inhibiting cytochrome P450 2C9
Source: PLoS Comput Biol. 2022 Jan 26;18(1):e1009820. doi: 10.1371/journal.pcbi.1009820 (PMC8820617; doi:10.1371/journal.pcbi.1009820)
Supplement: S1 Table — (PDF) [file pcbi.1009820.s009.pdf]

Table S1. Mean importance inferred from the random forest modeling of the best 36 physicochemical descriptors and 7 IEs discriminating between inhibitors and non-inhibitors of CYP2C9.

Categories	Descriptor name	Description	Importance
MOE 2D molecular descriptors			
Lipophilicity	SlogP	log of the octanol/water partition coefficient	131.48
	h_logD	log of the octanol/water partition coefficient at pH 7	124.92
	logP(o/w)	log of the octanol/water partition coefficient (including implicit hydrogens)	96.11
	BCUT_SLOGP_0	BCUT# descriptor using atomic contribution to logP	56.31
	GCUT_SLOGP_0	GCUT& descriptor using atomic contribution to logP	35.10
	GCUT_SLOGP_2	GCUT& descriptor using atomic contribution to logP	26.20
	SlogP_VSA9	van der Waals surface area for atoms contributing to SlogP >0.40	21.84
	SlogP_VSA3	van der Waals surface area for atoms contributing to SlogP within [0,0.1]	21.62
Polarity	PEOE_VSA_NEG	total negative van der Waals surface area calculated using partial charges calculated with the PEOE method	72.22
	PEOE_VSA-1	van der Waals surface of atoms with charge in the range [-0.10,-0.05) calculated with the PEOE method	32.02
	PEOE_VSA_FNEG	fractional negative van der Waals surface area calculated using the PEOE method	29.88
	Q_VSA_FNEG	fractional negative van der Waals surface area calculated using the partial charges	25.88

		stored with each structure in the database	
	Q_VSA_FPPOS	fractional positive polar van der Waals surface area	24.00
Charges	BCUT_PEOE_0	BCUT descriptor using PEOE partial charges	46.58
	BCUT_PEOE_3	BCUT descriptor using PEOE partial charges	34.95
	GCUT_PEOE_0	GCUT descriptor using PEOE partial charges	31.54
	BCUT_PEOE_2	BCUT descriptor using PEOE partial charges	22.10
Atom types and bounds	h_log_pbo	sum of log (1+pi bond order) for all bonds based on a modified Hueckel Theory calculation	74.77
	a_aro	number of aromatic atoms	51.38
	balabanJ	Balaban's connectivity topological index	23.82
Molar refractivity	SMR_VSA_5	accessible van der Waals surface area for atoms with Molar Refractivity in the range [0.44,0.485]	34.15
	GCUT_SMR_0	GCUT descriptor using atomic contribution to molar refractivity	31.37
	GCUT_SMR_2	GCUT descriptor using atomic contribution to molar refractivity	28.54
Functional descriptor<br><br>Rule of three	ast_violation	Astex Fragment-like Violation Count	37.97
MOE 3D molecular descriptors			
Volume and shape, packing	pmi1	first diagonal element of diagonalized moment of inertia tensor	40.39
	vsurf_R	surface rugosity	36.70
	std_dim2	the square root of the second largest eigenvalue covariance	23.04

		matrix of the atomic coordinates	
	vsurf_CP	critical packing parameter	22.03
Conformation dependent negative surface	ASA-	water accessible surface area of all atoms with negative partial charges	59.75
	CASA-	negative charge weighted surface area	32.68
Potential energy	E_oop	out-of-plane potential energy	48.95
	E_ang	angle bend potential energy	32.78
	AM1_E	the total SCF energy calculated using the MOPAC AM1 Hamiltonian	28.81
	E_str	bond stretch potential energy	27.43
	E	potential energy	22.53
Interaction energies (IE)	IE1<br>IE2<br>IE3<br>IE4<br>IE5<br>IE6<br>IE7	IE computed on MD1<br>IE computed on MD2<br>IE computed on 1R9O<br>IE computed on MD3<br>IE computed on 5XXI<br>IE computed on MD4<br>IE computed on MD5	33.84<br>32.75<br>30.79<br>29.89<br>28.20<br>26.03<br>22.32

#The BCUT descriptors [1] were calculated from the eigenvalues of a modified adjacency matrix (e.g., the diagonal takes PEOE partial charges or atomic contribution to logP). Each ij entry of the adjacency matrix takes the value of 1/sqrt(bij), where bij is the formal bond order between bonded atoms i and j.
&The GCUT descriptors were calculated from the eigenvalues of a modified graph distance adjacency matrix (e.g., the diagonal takes PEOE partial charges or atomic contribution to logP). The MD structures correspond to MD simulations of CYP2C9 apo, CYP2C9 bound to diclofenac, or CYP2C9 bound to losartan: MD1, 0003_apo; MD2, 0000_diclofenac; MD3, 0002_losartan; MD4, 0000_losartan; and MD5, 0002_WT_apo.

References

1. Pearlman RS, Smith KM. Novel software tools for chemical diversity. Perspectives in Drug Discovery and Design. 1998; 9:339–353
