# Supplementary material for: Machine learning-driven identification of drugs inhibiting cytochrome P450 2C9
Source: PLoS Comput Biol. 2022 Jan 26;18(1):e1009820. doi: 10.1371/journal.pcbi.1009820 (PMC8820617; doi:10.1371/journal.pcbi.1009820)
Supplement: S2 Table — (PDF) [file pcbi.1009820.s010.pdf]

Table S2. Performances of preliminary RF models with the best MOE descriptors on the training set.

**Descriptors**	10 MOE	15 MOE	20 MOE	30 MOE	40 MOE
**Balanced accuracy %**	72.27	75.57	78.61	79.43	77.80
**Sensitivity %**	77.45	75.46	79.82	81.57	78.98
**Specificity %**	67.08	75.67	77.39	77.29	76.62
