# Supplementary material for: Machine learning-driven identification of drugs inhibiting cytochrome P450 2C9
Source: PLoS Comput Biol. 2022 Jan 26;18(1):e1009820. doi: 10.1371/journal.pcbi.1009820 (PMC8820617; doi:10.1371/journal.pcbi.1009820)
Supplement: S3 Table — (PDF) [file pcbi.1009820.s011.pdf]

Table S3. Parameters of the optimized best RF and SVM models.

Best models	15 MOE +7 IE descriptors	20 MOE +7 IE descriptors	36 MOE +7 IE descriptors	170 MOE +7 IE descriptors
**RF *ntree***	250	300	275	275
**RF *mtry***	7	7	11	9
**SVM Cost**	8	4	8	4
**SVM Sigma**	0.0452	0.0348	0.0203	0.0036
