# Supplementary material for: Machine learning-driven identification of drugs inhibiting cytochrome P450 2C9
Source: PLoS Comput Biol. 2022 Jan 26;18(1):e1009820. doi: 10.1371/journal.pcbi.1009820 (PMC8820617; doi:10.1371/journal.pcbi.1009820)
Supplement: S4 Table — (PDF) [file pcbi.1009820.s012.pdf]

Table S4. Performances of the optimized RF and SVM models with MOE descriptors on the external validation set.

Descriptors	Models	Accuracy %	Sensitivity %	Specificity %	MCC %
15 MOE	RF<br>SVM	83.16<br>82.49	86.66<br>86.76	78.65<br>76.97	67.36<br>65.89
20 MOE	RF<br>SVM	83.39<br>82.09	86.56<br>86.26	79.30<br>76.71	68.27<br>68.11
36 MOE	RF<br>SVM	83.95<br>82.66	87.06<br>87.36	79.95<br>76.58	69.47<br>66.52
170 MOE	RF<br>SVM	84.18<br>84.75	87.96<br>88.47	79.30<br>79.95	70.85<br>70.32
