# Supplementary material for: Machine learning-driven identification of drugs inhibiting cytochrome P450 2C9
Source: PLoS Comput Biol. 2022 Jan 26;18(1):e1009820. doi: 10.1371/journal.pcbi.1009820 (PMC8820617; doi:10.1371/journal.pcbi.1009820)
Supplement: S5 Table — (PDF) [file pcbi.1009820.s013.pdf]

Table S5. Comparison of datasets used and performances of models as reported in the literature.

Models	Validation	Balanced accuracy %	Sensitivity %	Specificity %	MCC %	Number of inhibitors/non-inhibitors
Cheng et al. [1]<br>*Model CC-III*	Training CV<br>External set	74.25<br>76.10	63.90<br>56.20	84.60<br>96.00	49.60<br>59.20	4369/7761<br>609/1970
Li et al. [2]<br>*Multitask model*	Training set<br>External set	84.20<br>71.05	80.80<br>52.20	87.60<br>89.90	65.80<br>36.50	2552/6833<br>69/596
Wu et al. [3]<br>*XGBoost model*	Training set<br>External set	83.15<br>63.15	73.30<br>29.00	93.00<br>97.30	68.00<br>35.40	2552/6833<br>69/596
Racz et al. [4]<br>*Model 1*	Training CV<br>External set	80.50<br>79.00	82.00<br>82.00	79.00<br>76.00	0.61<br>0.48	6488/7617<br>2209/2593
RF<br>***36 MOE + 7 IE***	Training CV<br>External set	82.45<br>84.33	89.13<br>89.97	75.76<br>78.69	65.96<br>69.45	3872/2641<br>968/660
SVM<br>***36 MOE + 7 IE***	Training CV<br>External set	81.41<br>83.35	87.68<br>89.87	75.13<br>76.83	63.62<br>67.72	3872/2641<br>968/660

References

1. Cheng F, Yu Y, Shen J, Yang L, Li W, Liu G, et al. Classification of cytochrome P450 inhibitors and noninhibitors using combined classifiers. J Chem Inf Model 2011;51:996–1011.
2. Li X, Xu Y, Lai L, Pei J. Prediction of human cytochrome P450 inhibition using a multitask deep autoencoder neural network. Mol Pharm 2018;15:4336–45.
3. Wu Z, Lei T, Shen C, Wang Z, Cao D, Hou T. ADMET evaluation in drug discovery. 19. Reliable prediction of human cytochrome P450 inhibition using artificial intelligence approaches. J Chem Inf Model. 2019;59:4587–601.
4. Racz A, Keseru GM. Large-scale evaluation of cytochrome P450 2C9 mediated drug interaction potential with machine learning-based consensus modeling . J Comput Aided Mol Des. 2020;34:831-9.
