# Supplementary material for: Machine learning-driven identification of drugs inhibiting cytochrome P450 2C9
Source: PLoS Comput Biol. 2022 Jan 26;18(1):e1009820. doi: 10.1371/journal.pcbi.1009820 (PMC8820617; doi:10.1371/journal.pcbi.1009820)
Supplement: S6 Table — (PDF) [file pcbi.1009820.s014.pdf]

Table S6. List of the experimentally tested drugs.

Diversity Cluster	Drug	Structure	ZINC entry<br>Provider
1	Abemaciclib	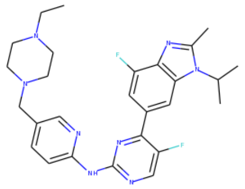	ZINC000072318121<br>CliniSciences, France
2	Mizolastine	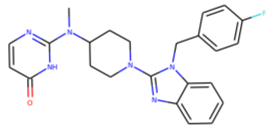	ZINC000013831810<br>Bertin Bioreagent, France
3	Sertindole	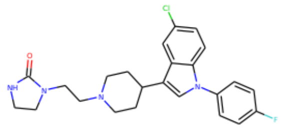	ZINC000000538337<br>CliniSciences, France
3	Cloperidone	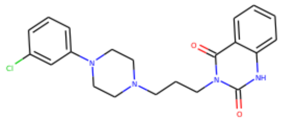	ZINC000004215426<br>Ambinter, France
5	Sivelestat	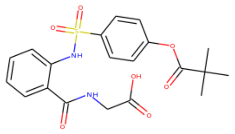	ZINC000021298097<br>Bertin Bioreagent, France
6	Asapiprant	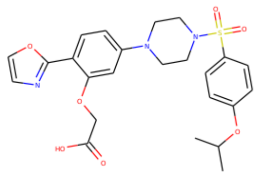	ZINC000115250035<br>CliniSciences, France
7	Pf-562671	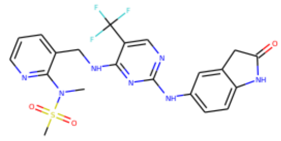	ZINC000034638188<br>Bertin Bioreagent, France
8	Ciltoprazine	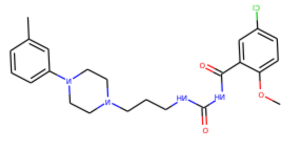	ZINC000004215333<br>BOC Sciences, NY, USA

10	Vatalanib	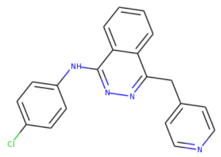	ZINC000000007460<br>Sigma Aldrich, France
11	Entinostat	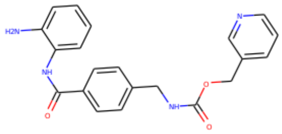	ZINC000001488870<br>Bertin Bioreagent, France
12	Azd3514	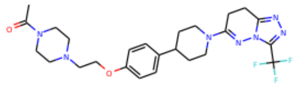	ZINC000101673084<br>Bertin Bioreagent, France
14	Muraglitazar	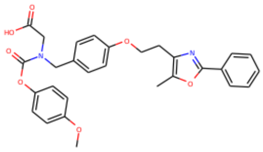	ZINC000049650290<br>Bertin Bioreagent, France
16	Bifeprofen	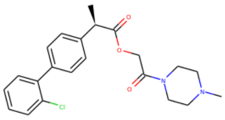	ZINC000031502465<br>BOC Sciences, NY, USA
22	Tarafenacin	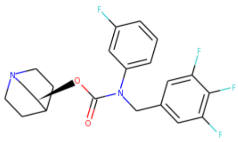	ZINC000000593626<br>CliniSciences, France
23	Ticagrelor	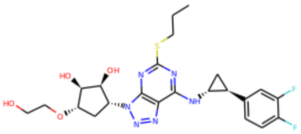	ZINC000028957444<br>Bertin Bioreagent, France
24	Duvelisib	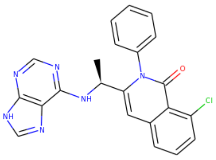	ZINC000088346058<br>Bertin Bioreagent, France
24	Dasatinib	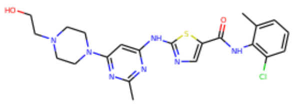	ZINC000003986735<br>CliniSciences, France
24	Piriqualone	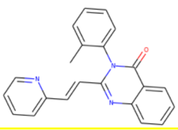	ZINC000004217322<br>Mcule, Hungary
